# Review of the millipede genus *Orthomorpha* Bollman, 1893 (Diplopoda, Polydesmida, Paradoxosomatidae) in Vietnam, with several new records and descriptions of two new species

**DOI:** 10.3897/zookeys.898.39265

**Published:** 2019-12-10

**Authors:** Natdanai Likhitrakarn, Sergei I. Golovatch, Irina Semenyuk, Boris D. Efeykin, Somsak Panha

**Affiliations:** 1 Division of Plant Protection, Faculty of Agricultural Production, Maejo University, Chiang Mai, 50290, Thailand Maejo University Chiang Mai Thailand; 2 Severtsov Institute for Problems of Ecology and Evolution, Russian Academy of Sciences, Leninsky pr. 33, Moscow, 119071, Russia Severtsov Institute for Problems of Ecology and Evolution, Russian Academy of Sciences Moscow Russia; 3 Joint Russian-Vietnamese Tropical Center, Street 3 tang 2, 3, q10, Ho Chi Minh City, Vietnam Joint Russian-Vietnamese Tropical Center Ho Chi Minh Vietnam; 4 Kharkevich Institute for Information Transmission Problems, Russian Academy of Sciences, Bol’shoi Karetnyi per. 19, Moscow, 127051, Russia Russian Academy of Sciences Moscow Russia; 5 Animal Systematics Research Unit, Department of Biology, Faculty of Science, Chulalongkorn University, Bangkok, 10330, Thailand Chulalongkorn University Bangkok Thailand

**Keywords:** taxonomy, variety, barcoding, phylogeny, key, map

## Abstract

The genus *Orthomorpha* is shown to currently be represented in Vietnam by ten species or varieties, including new records of *O. arboricola* (Attems, 1937), *O. coarctata* (de Saussure, 1860), *O. rotundicollis* (Attems, 1937) and *O. scabra* Jeekel, 1964, and two new species, *O. caramel***sp. nov.** and *O. vietnamica***sp. nov.** A key to all eight *Orthomorpha* species and two varieties known to occur in Vietnam is provided. Although the morphological characters that have been traditionally used for *Orthomorpha* taxonomy are here considered superior to molecular ones, molecular-based phylogenetic relationships and taxon assignments within the tribe Orthomorphini are provisionally analyzed using fragments of the cytochrome c oxidase subunit I (COI) mitochondrial gene. The preferred phylograms, both rooted and unrooted, demonstrate the monophyly of the tribe Orthomorphini, but due to the special, uncertain or even controversial position of *O. coarctata*, which occurs closer to the genera *Antheromorpha* and *Hylomus*, the genus *Orthomorpha* in current usage appears to be polyphyletic. However, if *O. coarctata* is to be treated within the monotypic genus *Asiomorpha*, the monophyly of *Orthomorpha* becomes manifest. On the other hand, a cautious approach is followed to avoid descriptions of suspicious new taxa/species. Thus, solely because the average genetic distance between *O. rodundicollis subrotundicollis***var. nov.** and *O. rodundicollis*, as well as that between *O. scabra grandis***var. nov.** and *O. scabra*, are both found to be negligibly small, the statuses of the sympatric and closest yet morphologically different varieties are treated only as such, i.e., infrasubspecific categories. The apparent discord observed between morphological and molecular data is obviously due to only partial and single-gene topologies used, possibly also to hybridization already known to occur in some closely related and sympatric paradoxosomatid species or even genera.

## Introduction

The Southeast Asian millipede genus *Orthomorpha* Bollman, 1893 is the largest in the tribe Orthomorphini, family Paradoxosomatidae (Nguyen and Sierwald 2013). This genus has recently been reviewed and shown to comprise as many as 54 species ranging from northern Myanmar and Thailand in the northwest to Lombok Island, Indonesia in the southeast (Likhitrakarn et al. 2011, 2014). Only one species, *O. coarctata* (de Saussure, 1860), has attained a pantropical distribution due to anthropochory.

Only six recognized species of *Orthomorpha* have hitherto been known to occur in Vietnam. Among them, *O. hydrobiologica* (Attems, 1937), *O. rotundicollis* (Attems, 1937), and *O. scabra* Jeekel, 1964 are widespread also in Laos and Cambodia (Likhitrakarn et al. 2011, 2013, 2014).

This paper is devoted to descriptions of two new species from southern Vietnam, based on the material collected by one of us (IS) in the Cat Tien, Kon Ka Kinh, Hon Ba and Bidoup Nui Ba national parks, all being priority zones for the protection of biodiversity in Vietnam (e.g., Polet and Ling 2004). In addition, we provide new records of three previously described species and a key to all eight Vietnamese species of the genus, with their distributions mapped.

Molecular analyses were performed to obtain a phylogram in order to visualize the phylogenetic relationships. The topology from MrBayes was consistent with the present-day morphology-based taxonomy/classification of the tribe Orthomorphini (Nguyen and Sierwald 2013).

## Materials and methods

The specimens were collected as part of IS’ research project on the diversity, biology and ecology of millipedes in Vietnam. Field-work by IS, including material collection, was conducted in accordance with Agreement 37/HD for the scientific cooperation between the Cat Tien National Park and the Joint Russian-Vietnamese Tropical Center; that in the Kon Ka Kinh National Park according to Agreements 432/TCLN-BTTN and 142/SNgV-VP; and that in the Bidoup Nui Ba National Park and Hon Ba Nature Reserve according to Agreement 774/TCLN-DDPH. The Animal Care and Use Protocol Review No. 1723018 was strictly followed. Coordinates and elevations were recorded by Garmin eTrex 30 using the WGS84 datum and subsequently double-checked with Google Earth.

Live animals were photographed in their habitats using a Canon PowerShot A4000IS 16.0 MP Digital Camera and Panasonic DMC-TZ80 – LUMIX Digital Camera. Specimens were preserved in 96% ethanol, and morphological characters were studied in the laboratory using an Olympus stereo microscope. Scanning electron micrographs (SEM) of coated gonopod specimens were taken using a JEOL, JSM–5410 LV microscope. Specimens were also photographed in the laboratory as digital images assembly using automontage software techniques, while gonopods were also illustrated. All material, including the holotypes and voucher specimens for molecular analyses, is housed in the collection of the Zoological Museum, State University of Moscow (**ZMUM**), Russia.

In the catalogue sections, D stands for the original description, subsequent descriptive notes or appearance in a key, R for a subsequent record or records, N for giving a new name while M for a mere mention and Di stands for genetic distances in the molecular analyses.

DNA was isolated from specimens fixed in 96% ethanol using QiaAMP DNA Mini Kit (Qiagen). Sequences of the cytochrome oxidase subunit I (COI) gene were amplified using an EncycloPlus PCR kit (Evrogen, Russia) with the primer set COI-1F20 (5'-ACT CTA CTA ATC ATA AGG AT-3') and COI-1R19 (5'-TAA ACC TCC GGG TGA CCAA-3') derived from Nguyen et al. (2017). Polymerase chain reaction (PCR) products were visualized with the help of gel electrophoresis, excised, and cleaned using a SV Gel and PCR CleanUp System kit (Evrogen, Russia). The sequences were combined and aligned using ClustalX software after the addition of sequences from the GenBank (Thompson et al. 1997). MEGA 6.06 (Tamura et al. 2013) was used to calculate the genetic distances between samples. Of the trees in the post burn-in posterior distribution inferred with MrBayes version 3.2.3, a consensus tree was constructed using sumt (Huelsenbeck and Ronquist 2001). The MrBayes analysis was conducted in the CIPRES server with the evolutionary model selected based on the results of the analysis in jModelTest2 (Darriba et al. 2015). The sequences have been deposited in NCBI GenBank (Table 1). Unfortunately, as there were fixation problems with the DNA, only around a third of the COI gene became available for analyses.

**Table 1.** Species voucher repository and accession numbers deposited in GenBank.

№	**Species**	**Location**	**COI sequences numbers**	**Voucher repository**
1	*Orthomorpha coarctata*	Thac Mai	MC762236	ZMUM
2	Orthomorpha rotundicollis var. subrotundicollis	Kon Ka Kinh	MC762237	ZMUM Rd 4210
3	*Orthomorpha rotundicollis*	Thac Mai	MC762238	ZMUM Rd 4215
4	*Orthomorpha rotundicollis*	Cat Tien	MC762239	ZMUM
5	*Orthomorpha caramel*	Kon Ka Kinh	MC762240	ZMUM Rd 4198
6	*Orthomorpha vietnamica*	Kon Ka Kinh	MC762241	ZMUM Rd 4199
7	*Orthomorpha scabra*	Kon Ka Kinh	MC762242	ZMUM Rd 4213
8	Orthomorpha scabra var. grandis	Bidoup Nui Ba	MC762243	ZMUM Rd 4267
9	*Orthomorpha arboricola*	Hon Ba	MC762244	ZMUM

## Taxonomic part

### Family Paradoxosomatidae Daday, 1889

#### Tribe Orthomorphini Brölemann, 1916

**Genus *Orthomorpha* Bollman, 1893**

##### Orthomorpha arboricola

Taxon classificationAnimaliaPolydesmidaParadoxosomatidae

(Attems, 1937)

18200CCA-A9A7-5844-9398-1F373C4B79E1

Figs 1 , 2

Pratinus arboricola Attems, 1937: 120 (D).
Pratinus arboricola – Attems 1938: 222 (D).
Orthomorpha arboricola – Jeekel 1963: 265 (M); 1964: 361 (M, D); 1968: 56 (M); Enghoff et al. 2004: 38 (M); Golovatch 1998: 42 (D); Likhitrakarn et al. 2011: 18 (D).

###### Old records.

Vietnam, Lamdong Province, Dalat, 1,500 m a.s.l.; Lamdong Province, Langbian Mountain, Trąm Hành (= Arbre-Broyé) (Attems 1937, 1938).

###### New material examined.

2 ♂ (ZMUM), Vietnam, Khanh Hoa Province, Hon Ba Nature Reserve, 12°07'02"N, 108°56'45"E, 1,550 m a.s.l., mixed mossy forest on mountain ridge, on forest floor, night time, 27.VI.2018, I. Semenyuk leg.

###### Descriptive notes.

Length 40–41 mm (♂), width of midbody pro- and metazonae 2.7–2.8 and 4.3–4.4 mm (♂), respectively.

**Figure 1.**
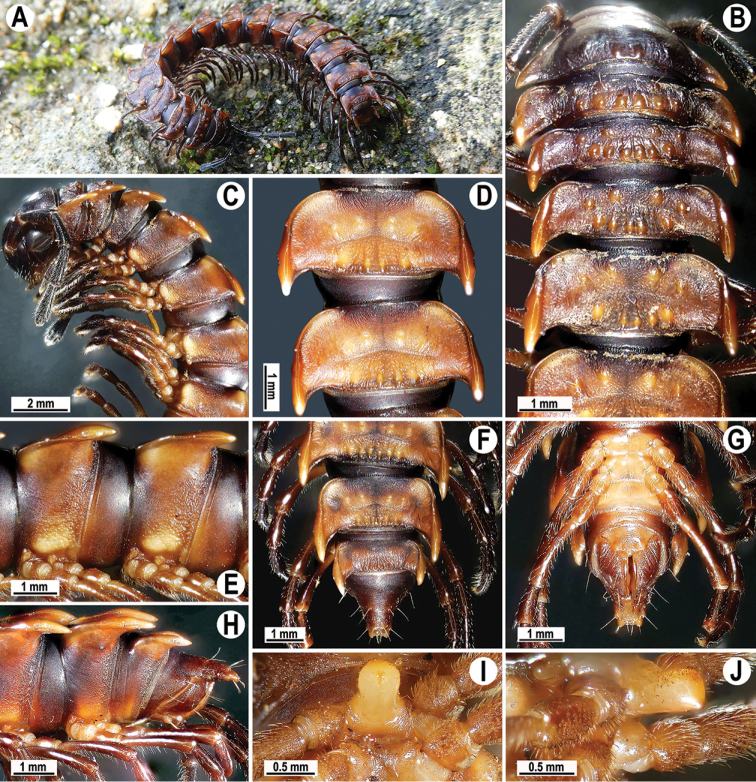
*Orthomorpha arboricola* (Attems, 1937), ♂ from Hon Ba National Park **A** habitus and live colouration **B, C** anterior part of body, dorsal and lateral views, respectively **D, E** segments 10 and 11, dorsal and lateral views, respectively **F–H** posterior part of body, dorsal, ventral and lateral views, respectively **I, J** sternal cone between coxae 4, subcaudal and sublateral views, respectively.

Live colouration (Fig. 1A) and colouration in alcohol, after one year of preservation, similar, only slightly faded in fixed material, yellow-brown to brown (Fig. 1B–F), paraterga, most of mid-dorsal parts of metaterga and tip of epiproct orange-yellow to light yellow, legs and sterna light dark brown to light yellowish, antennae blackish (Fig. 1A, B).

**Figure 2.**
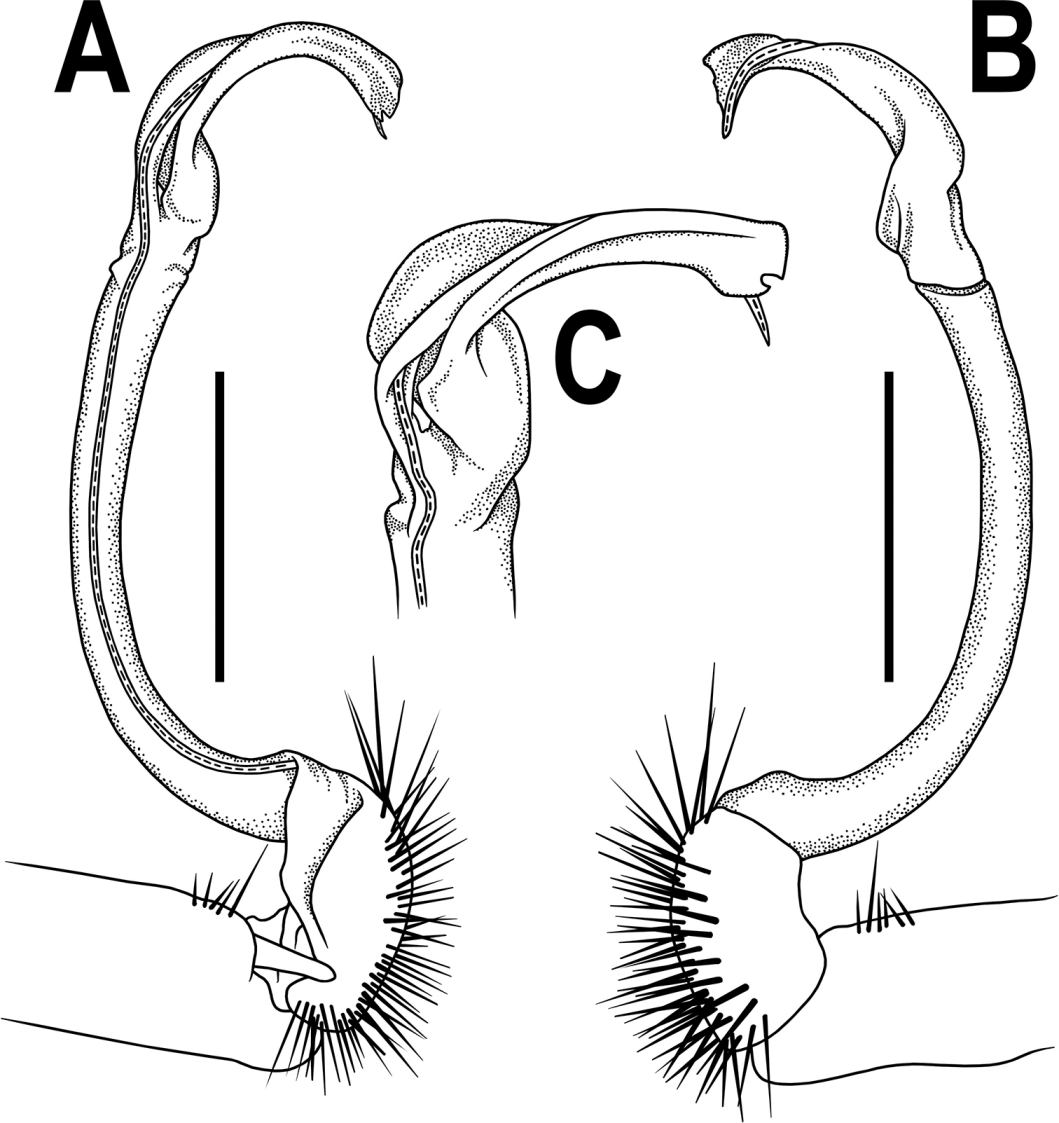
*Orthomorpha arboricola* (Attems, 1937), left gonopod **A, B** mesal and lateral views, respectively **C** tip of right gonopod, lateral view. Scale bars: 0.5 mm.

All characters as in the available descriptions (Attems 1937; Likhitrakarn et al. 2011), except as follows. Posterior (postsulcus) transverse row of 3(4)+3(4) low, setigerous, oblong, rounded tubercles on postcollum metaterga. A prominent, tongue-shaped, rounded, setose cone or lobe between ♂ coxae 4 (Fig. 1I, J). Pleurosternal carinae complete crests only on segments 2–4 (♂) (Fig. 1B, D), each with an evident sharp denticle caudally, thereafter increasingly reduced until segment 11. Solenophore tip very faintly bidentate, with terminal tooth bearing a minute denticle at base (Fig. 2); solenomere long and flagelliform, as usual in the genus.

###### Remarks.

This species is known not only from type material from southern Vietnam (Dalat and Peak Lang Biang, at both sites found to coexist with *O. rotundicollis* (Attems, 1937)), but also from the new samples cited above. The types have recently been redescribed by Likhitrakarn et al. (2011), while the above new material shows only minor variations.

##### Orthomorpha coarctata

Taxon classificationAnimaliaPolydesmidaParadoxosomatidae

(de Saussure, 1860)

690F9570-2FAB-55AB-B7AE-8502231F42B2

Polydesmus coarctatus de Saussure, 1860: 297 (D).
Paradesmus flavocarinatus Daday, 1889: 136 (D). Synonymized by Enghoff (2005).
Orthomorpha coarctata – Pocock 1895: 809 (R, M, K); Attems 1937: 62 (D); Jeekel 1968: 45 (M); Likhitrakarn et al. 2011: 12 (D, R, K), Golovatch and Wesener 2016: 47 (L); et auctorum.
Orthomorpha coarctata gigas Attems, 1927: 63 (D). Synonymized by Jeekel (1968).
Asiomorpha coarctata – Verhoeff 1939: 117 (D); Nguyen and Sierwald 2013: 1236 (L); et auctorum.
Orthomorpha coarctata gigas – Jeekel 1968: 45 (M); Golovatch 1998: 43 (K); et auctorum.

###### New material examined.

3 ♂, 1 ♀, 2 juv. (ZMUM), Vietnam, Dong Nai Province, Cat Tien National Park, 11°25'16"N, 107°25'39"E, 120 m a.s.l., on floor between huts in the Park’s headquarters, an open site in monsoon tropical forest, night time, 5 & 8.VIII.2015; 3 ♂, 2 ♀ (ZMUM), Vietnam, Dong Nai Province, Thac Mai waterfall and hot spring area, 11°06'12"N, 107°24'24"E, grassy recreation area near pond, in leaf litter, daytime, 01.06.2018, all I. Semenyuk leg.

###### Remarks.

This pantropical anthropochore species has been redescribed several times and recently revised (Likhitrakarn et al. 2011). It is often assigned to the monotypic genus *Asiomorpha* Verhoeff, 1939 (e.g., Nguyen and Sierwald 2013), but we follow Jeekel (1968) who treated it in *Orthomorpha*. Molecular evidence, however, shows a very strong genetic isolation of *coarctata* from the other formal congeners, thus favouring the recognition of *Asiomorpha* as a separate monobasic genus (Fig 20). This species has already been recorded from Vietnam, on sea shore north of Nhatrang (Golovatch 2017).

The new samples belong to the typical form of *O. coarctata*, whereas the only other form, *O. c. gigas* Attems, 1927, from Ambon, Indonesia, has long been synonymized under *O. c. coarctata* (see Jeekel 1968; Likhitrakarn et al. 2011).

##### Orthomorpha glandulosa

Taxon classificationAnimaliaPolydesmidaParadoxosomatidae

(Attems, 1937)

0262C6E5-6D30-5215-BED1-44520B38856A

Pratinus glandulosus Attems, 1937: 119 (D).
Pratinus glandulosus – Attems 1938: 220 (D).
Orthomorpha glandulosa – Jeekel 1963: 265 (M); 1964: 361 (M, D); 1968: 56 (M); Hoffman 1977: 700 (M); Golovatch 1998: 42 (D, M); Enghoff et al. 2004: 38 (M, R); Likhitrakarn et al. 2011: 72 (D).

###### Old records.

Vietnam, Dak Lak Province, near frontier with Cambodia; Khanh Hoa Province, Nhatrang, Suoidau; same Province, Hon Ba Mountain (Attems 1937, 1938).

###### Remarks.

This species has recently been redescribed, based only on the type material (Likhitrakarn et al. 2011).

##### Orthomorpha hydrobiologica

Taxon classificationAnimaliaPolydesmidaParadoxosomatidae

Attems, 1930

DC760E2A-1A52-5E08-A581-77A7B4C8ED2F

Orthomorpha hydrobiologica Attems, 1930: 120 (D).
Orthomorpha hydrobiologica – Attems 1937: 63 (D); 1938: 215 (R); Jeekel 1963: 265 (M); 1964: 361 (M, D); 1968: 45 (M); Hoffman 1973: 362 (M); 1977: 700 (M); Golovatch 1998: 42 (M); Enghoff et al. 2004: 38 (R); Likhitrakarn et al. 2011: 53 (D); 2015: 181 (R).
Oxidus hydrobiologicus – Chamberlin 1945: 10 (R).

###### Old records.

Vietnam, Quang Ninh Province, Hong Gai; Khanh Hoa Province, Nha Trang; Cau Da; Ba Ngoi; Ninh Thuan Province, Phan Rang; Binh Phuoc Province, Loc Ninh; Dak Lak Province, near frontier with Cambodia; Kien Giang Province, Poulo Dama (Phu Quoc Island) (Attems 1938); Cambodia, Sihanoukville Province, Ream; Kampot Province, Phnom Bokor (Attems 1938); Indonesia, East Java Province, Lumajang Regency, shore of Ranu (= lake) Bedali (Attems 1930); West Java, Mt Pangrango; Krakatau; Karimon Djawa Island (Chamberlin 1945).

###### Remarks.

This species has recently been redescribed (Likhitrakarn et al. 2011), based both on type and non-type material. The species is widespread, probably due to anthropochory, especially along the coastal areas ranging from northern Vietnam down to southern Cambodia.

##### Orthomorpha rotundicollis

Taxon classificationAnimaliaPolydesmidaParadoxosomatidae

(Attems, 1937)

0686CEAA-93F2-5642-9EE0-690F509C7480

Figs 3 , 4 , 5

Pratinus rotundicollis Attems, 1937: 118 (D).
Pratinus rotundicollis – Attems 1938: 217 (D); 1953: 179 (R).
Pratinus tuberculatus Attems, 1937: 119 (D). Synonymized by Likhitrakarn et al. (2011).
Pratinus tuberculatus – Attems 1938: 219 (D).
Orthomorpha rotundicollis – Jeekel 1963: 265 (M); 1964: 361 (M, D); 1968: 56 (M); Hoffman 1973: 363 (M); 1977: 700 (M); Golovatch 1998: 42 (M, D); Enghoff et al. 2004: 38 (M, R); Likhitrakarn et al. 2011: 61 (D).
Orthomorpha tuberculata – Jeekel 1963: 265 (M); 1964: 361 (M, D); 1968: 56 (M); Hoffman 1977: 700 (M); Golovatch 1998: 42 (M, D); Enghoff et al. 2004: 39 (M, R).

###### Old records.

Vietnam, Lam Dong Province, Lang Biang Mountain, Tram Hanh (=Arbre-Broye), 1,500 m; Dalat, 1,500 (Attems 1937, 1938); Laos, Xiangkhoang Province, Xiangkhoang Plateau, Xiang Kuang (Attems 1953).

###### New material examined.

1 ♂ (yellow morph, ZMUM Rd 4215), Vietnam, Dong Nai Province, Cat Tien National Park, 11°25'37"N, 107°25'39"E, 140 m a.s.l., lowland monsoon tropical forest with dominating *Lagerstroemia calyculata* and *Afzelia xylocarpa*, on bush, daytime, 7.X.2016; 1 ♂ (yellow morph, ZMUM Rd 4217), same locality, under log, daytime, 6.V.2015; 1 ♂ (red morph, ZMUM Rd 4218), same locality, on forest floor, daytime, 10.V.2015; 1 ♂ (red morph, ZMUM Rd 4221), same locality, on bush, daytime, 3.VI.2015; 1 ♂ (red morph, ZMUM Rd 4222), same locality, on tree trunk, night time, 29.06.2015; 1 ♂ (red morph, ZMUM), same locality, hilly area with mainly bamboo forest, 11°22'50"N, 107°13'16"E, 110 m a.s.l., in leaf litter, daytime, 29.VI.2017; 1 ♂ (red morph, ZMUM), same locality but light open forest with highly dominating *L. calyculata*, 11°24'51"N, 107°22'34"E, 130 m a.s.l., on tree trunk, daytime, 20.09.2016; 1 ♂ (red morph, ZMUM), same locality, 11°27'09"N, 107°21'57"E, lava cave, on excrements of *Hipposideros* sp.; 2 ♂, 1 ♀ (red morph, ZMUM Rd 4216), same locality, on concrete floor between huts in headquarters of National Park, 11°25'16"N, 107°25'39"E, 120 m a.s.l., night time, 4.I.2015; 1 ♀ (red morph, ZMUM Rd 4219), same locality, 11°25'37"N, 107°25'39"E, 140 m a.s.l., lowland monsoon tropical forest with dominating *Lagerstroemia calyculata* and *Afzelia xylocarpa*, on tree trunk, daytime, 20.V.2015; 1 ♀ (yellow morph, ZMUM), same locality, on bush, night time, 14.VI.2015; 1 juv. (ZMUM Rd 4223), same locality, on tree trunk, night time, 9.VII.2015; 4 ♂, 2 ♀ (white form, ZMUM), Vietnam, Dong Nai Province, Thac Mai waterfall and hot spring area, 11°06'12"N, 107°24'24"E, grassy recreation area near pond, in leaf litter, daytime, 01.VI.2018, all I. Semenyuk leg.

###### Descriptive notes.

Length 29.0–38.5 mm (♂) or 31.5–40.5 mm (♀), width of midbody pro- and metazonae 2.3–2.8 and 3.5–4.3 mm (♂) or 2.8–3.3 and 3.6–4.9 mm (♀), respectively.

**Figure 3.**
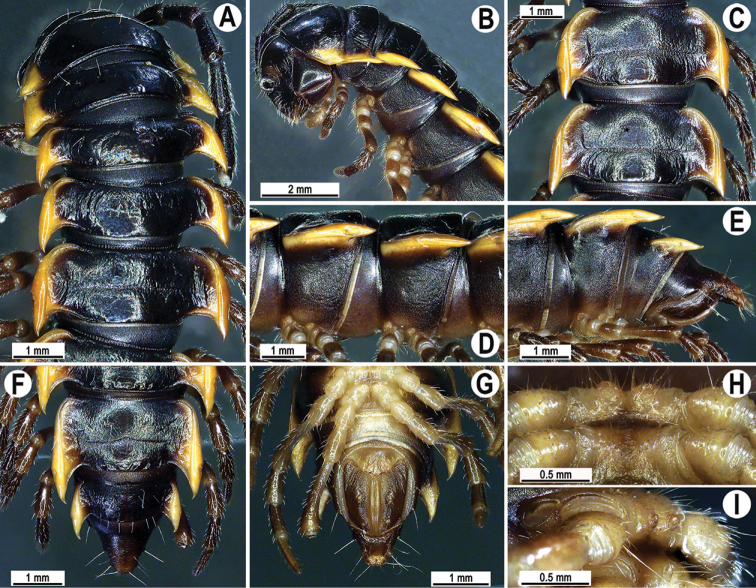
*Orthomorpha rotundicollis* (Attems, 1937), ♂ from Cat Tien National Park **A, B** anterior part of body, dorsal and lateral views, respectively **C, D** segments 10 and 11, dorsal and lateral views, respectively **E–G** posterior part of body, lateral, dorsal and ventral views, respectively **H, I** sternal cones between coxae 4, subcaudal and sublateral views, respectively.

Colouration in alcohol, after one year of preservation, blackish to black-brown (Fig. 3A–F), paraterga and epiproct orange-yellow to light yellow; legs and sterna light yellow to light brown; antennae yellowish to dark brownish distally (Fig. 3A). Colour polymorphism evident, colouration of paraterga generally ranging from white or yellow to red in one and the same population (see also below under Remarks).

Antennae rather long (Fig. 3A), projecting behind (♂) or reaching (♀) body segment 3 when stretched dorsally. Collum with caudal corner of paraterga dentiform, pointed, directed caudally, but not drawn past rear margin. Paraterga 2 broad, anterior edge rounded, lateral edge with two small incisions in anterior 1/3. Lateral edge of following paraterga with a small incision in anterior 1/3 (Fig. 3A, C, F) and caudal corner fully pointed, beak-like and slightly curved mesad, strongly produced behind rear tergal margin (Fig. 3A–G). Pleurosternal carinae complete crests with a sharp caudal tooth on segments 2–4, followings segments 5–10 each with an evident sharp denticle caudally, the latter gradually reduced to a small tooth until segment 15 (♂, ♀) (Fig. 3B, D, E). Epiproct (Fig. 3E–G) with evident apical papillae directed ventrocaudally.

Legs long and slender, midbody ones ca. 1.3–1.4 (♂) or 0.9–1.0 (♀) times as long as body height, ♂ tarsal brushes present on legs of segments 2–10, thereafter gradually thinning out until segment 16.

**Figure 4.**
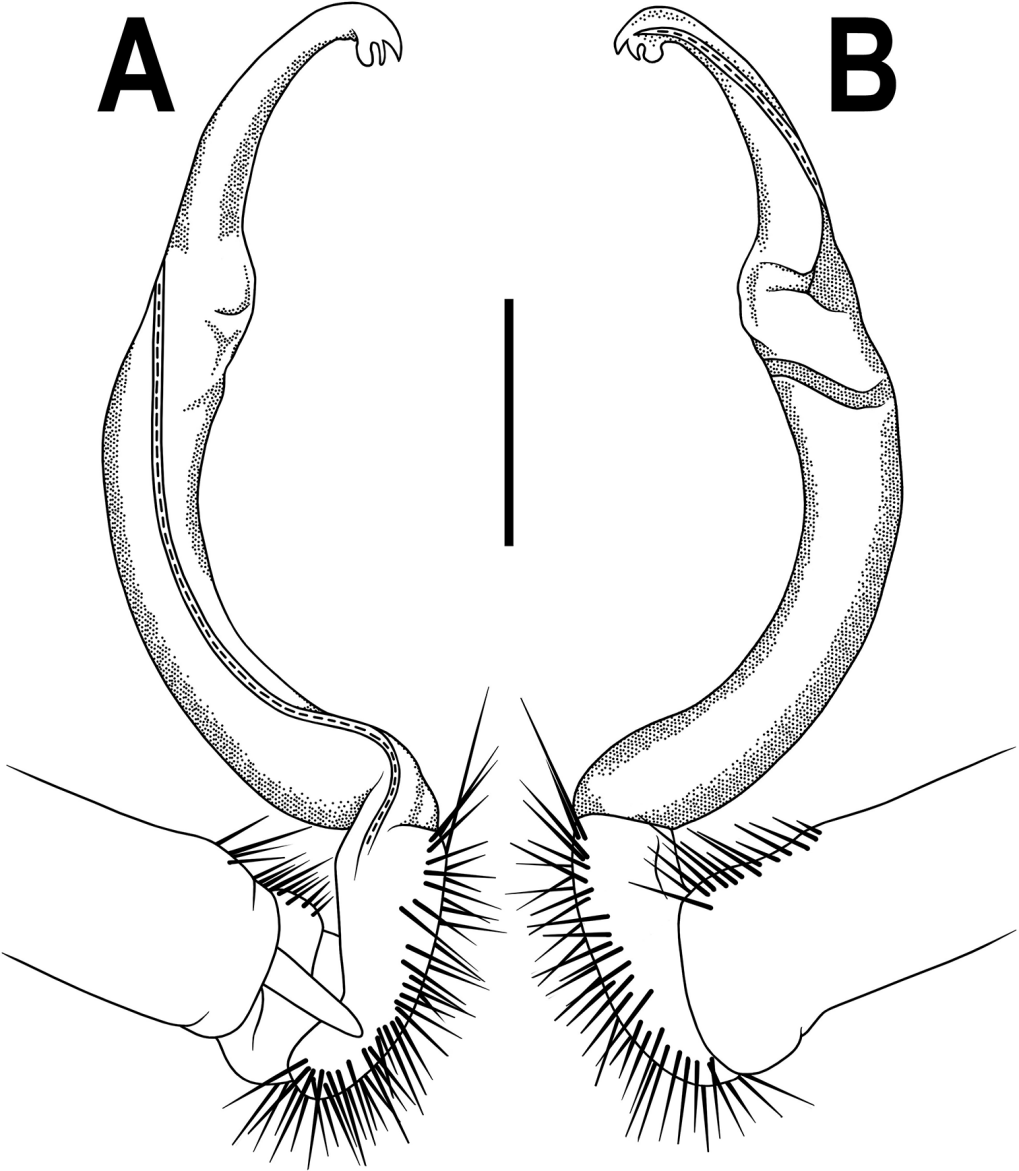
*Orthomorpha rotundicollis* (Attems, 1937), left gonopod **A, B** mesal and lateral views, respectively. Scale bar: 0.5 mm.

###### Remarks.

This species has recently been redescribed (Likhitrakarn et al. 2011), based on type material, and it is widespread from Luang Prabang Province, northern Laos to Cat Tien National Park, southern Vietnam (Fig. 19). The fresh specimens agree nearly fully with the available descriptions, except for the ♂ tarsal brushes being present until legs 10–14 vs. legs 5, and the pleurosternal carinae represented by small teeth gradually reduced until segment 15. These variations, as well as colour morphs, are certainly not more than infraspecific (cf. Likhitrakarn et al. 2011). One variety, *subrotundicollis* var. nov., is more disjunct morphologically, but not genetically, and is treated separately below.

The biology and behaviour of this species, referred to as *Orthomorpha* sp., has recently been described in detail in Cat Tien National Park (Semenyuk and Tiunov 2018). Millipedes are abundant almost throughout the year with a slight decline in the dry season (winter and early spring). Juveniles start swarming in the rainy season, mainly on logs or other decaying wood debris, also in suspended soil in tree holes. Swarms are active mostly in the night but can often be seen also in the daytime. Juveniles of later instars do not swarm, but still tend to group.

**Figure 5.**
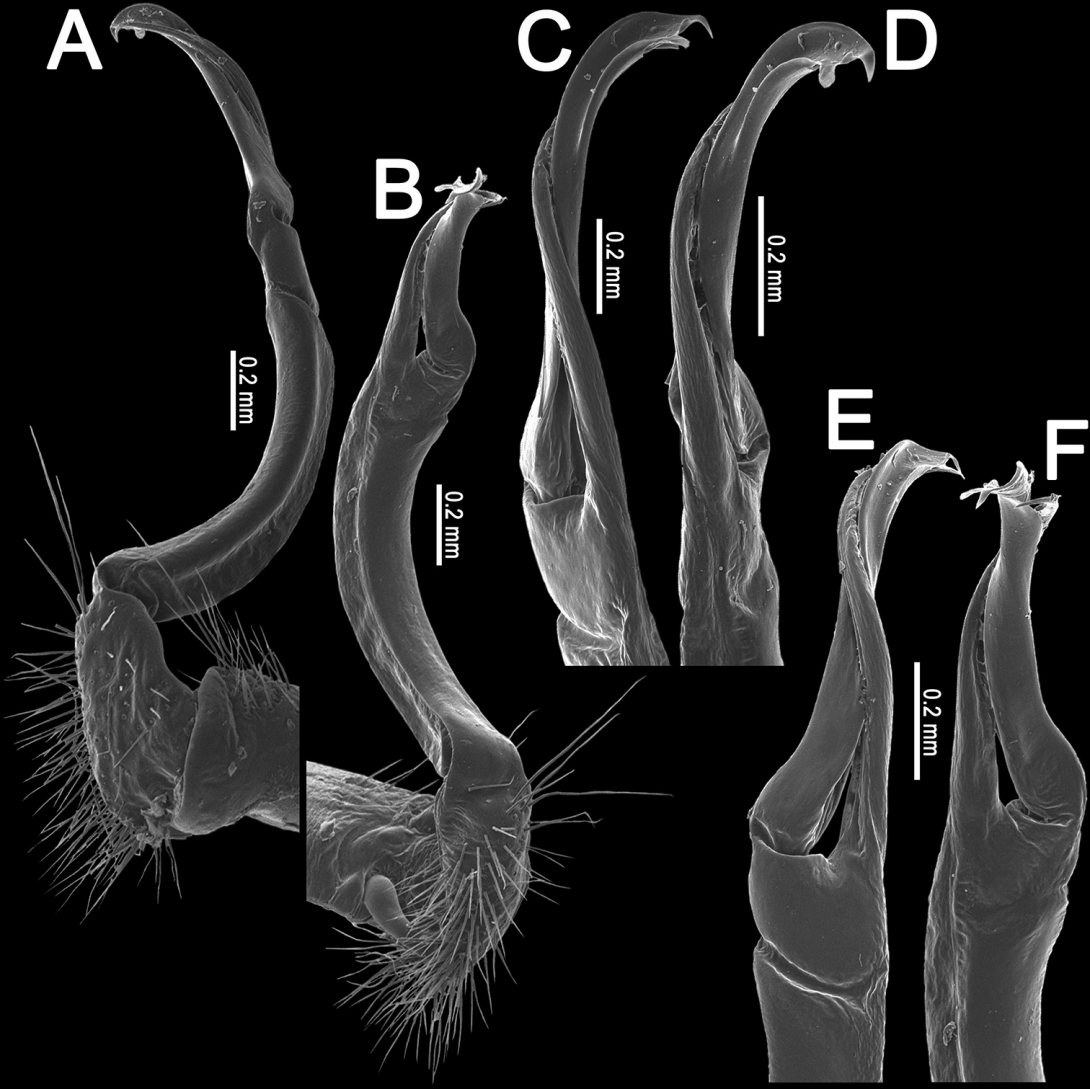
*Orthomorpha rotundicollis* (Attems, 1937), left gonopod **A, B** lateral and mesal views, respectively **C–F** distal part, suboral, oral, sublateral and submesal views, respectively.

##### Orthomorpha scabra

Taxon classificationAnimaliaPolydesmidaParadoxosomatidae

Jeekel, 1964

4BDFE925-C1D4-5CD2-9537-931853201D5D

Figs 6 , 7 , 8

Pratinus granosus Attems, 1953: 166 (D).
Orthomorpha granosa – Jeekel 1963: 265 (M).
Orthomorpha scabra Jeekel, 1964: 361 (N, D). Renamed by Jeekel (1964) to avoid homonymy.
Orthomorpha scabra – Jeekel 1968: 56 (M); Hoffman 1977: 700 (M); Golovatch 1998: 42 (M, D); Likhitrakarn et al. 2011: 58 (D).

###### Old records.

Vietnam, Lam Dong Province, Peak Lang Biang; Laos, Luang Prabang Province, Luang Prabang; Xiang Khouang Province, Xieng Khouang (Attems 1953).

###### New material examined.

1 ♂ (ZMUM Rd 4202), Vietnam, Gia Lai Province, Kon Ka Kinh National Park, forest around headquarters, 14°13'12"N, 108°19'54"E, 1,400 m a.s.l., mixed tropical forest on hill slope, under log, daytime, 26.V.2017; 2 ♂ (ZMUM Rd 4213), same locality, but near the village of Krong, 14°17'46"N, 108°26'41"E, 800 m a.s.l., disturbed tropical forest on hills with bamboo, on bushes, daytime, 18.V.2017; 3 ♀ (ZMUM), same locality, 14°18'59"N, 108°26'26"E, 750 m a.s.l., mixed tropical forest on slopes, on tree trunk, daytime, 9.05.2017; 2 ♂ (ZMUM), Vietnam, Lam Dong Province, Bidoup Nui Ba National Park, forest near Giang Ly ranger station, 12°10'23"N, 108°40'59"E, 1,550 m a.sl., mixed forest on slopes, in *Asplenium nidus*, daytime, 11.I.2018; 2 ♀ (ZMUM), same locality, mixed forest, 12°11'59"N, 108°40'34"E, 1,500 m a.s.l., on log, daytime, 13.I.2018; 3 ♂, 2 ♀ (ZMUM), same locality, 12°11'09"N, 108°40'43"E, 1,430 m a.s.l., mixed forest in the river valley, on forest floor, night time, 12 & 14.VI.2018, all leg. I. Semenyuk.

###### Descriptive notes.

Length 26–46 mm (♂) or 21.5–42 mm (♀), width of midbody pro- and metazonae 2.4–4.0 and 3.6–5.8 mm (♂) or 3.5–4.2 and 3.7–6.2 mm (♀), respectively.

**Figure 6.**
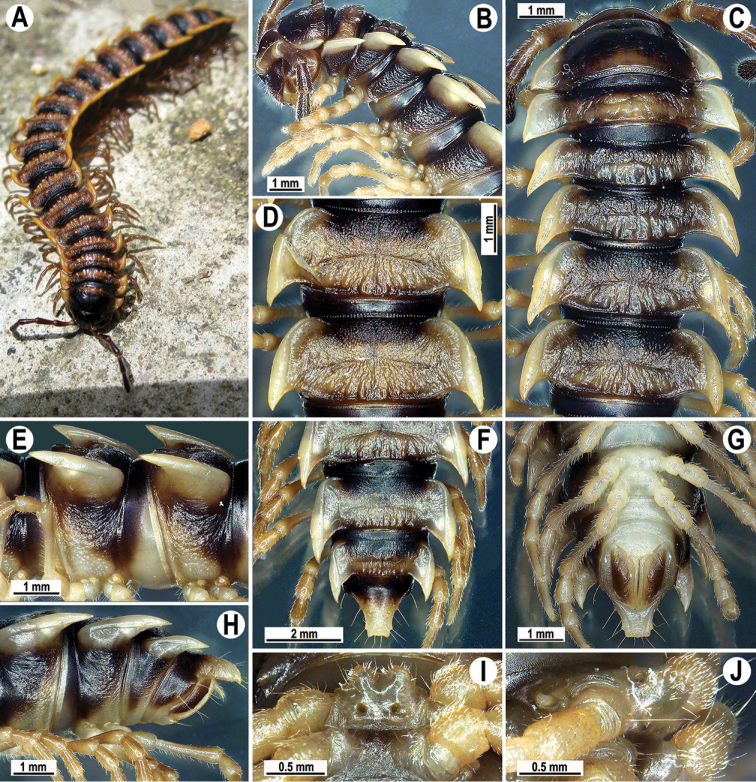
*Orthomorpha scabra* Jeekel, 1964, ♂ from Kon Ka Kinh National Park **A** habitus and live colouration **B, C** anterior part of body, lateral and dorsal views, respectively **D, E** segments 10 and 11, dorsal and lateral views, respectively **F–H** posterior part of body, dorsal, ventral and lateral views, respectively **I, J** sternal cones between coxae 4, subcaudal and sublateral views, respectively.

Colouration of live animals (Fig. 6A) black-brownish with contrasting dark yellow to orange paraterga and epiproct, head and antennae brownish, legs pale brownish; colouration in alcohol, after one year of preservation, faded to black-brown (Fig. 6B–H), paraterga and epiproct pale whitish yellow or pale brown, legs whitish to pale brown distally.

###### Remarks.

This species has recently been redescribed (Likhitrakarn et al. 2011), based on type material. The new specimens agree nearly fully with the available descriptions except for the ♂ tarsal brushes being present until legs 7 or 8 vs. legs 5. One variety, *grandis* var. nov., is more disjunct morphologically, but not genetically, being treated separately below.

**Figure 7.**
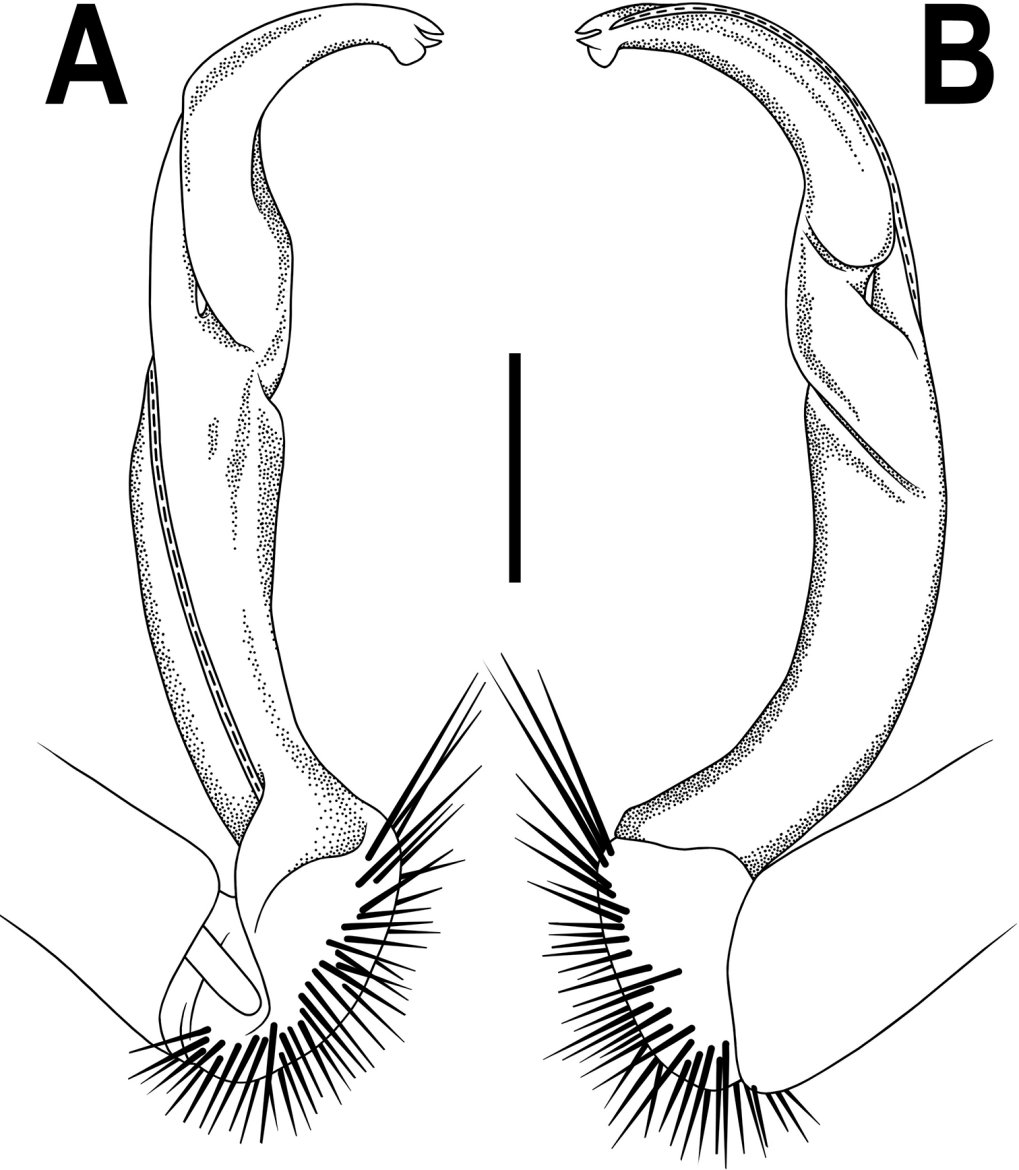
*Orthomorpha scabra* Jeekel, 1964, left gonopod **A, B** mesal and lateral views, respectively. Scale bar: 0.5 mm.

This species seems to be widespread, occurring in Vietnam and northern Laos (Fig. 19), and in two places it was found coexisting with *O. rotundicollis* (Attems, 1937) (see also above).

According to IS’ field observations, the millipedes occupy a wide range of habitats, including riparian forests with flooding occurring every year and non-flooding mixed forests on slopes. They also appear in a forestless farm area, usually being found on the forest floor and at bases of tree trunks. They occupy many microhabitats in lower forest strata. The activity is mainly restricted to the night time, but some individuals keep it up in daylight as well.

**Figure 8.**
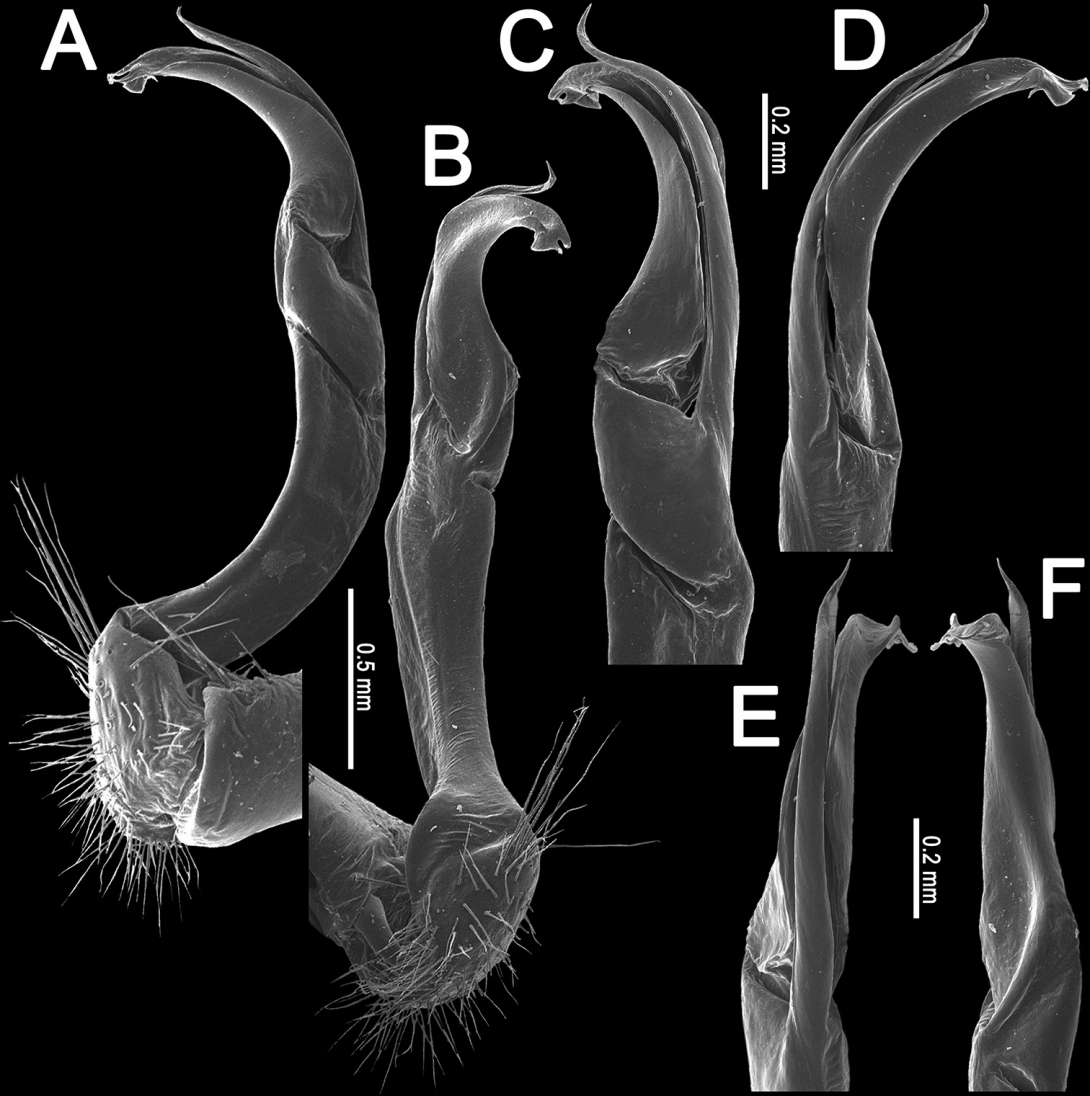
*Orthomorpha scabra* Jeekel, 1964, left gonopod **A, B** lateral and mesal views, respectively **C–F** distal part, sublateral, subcaudal, caudal and oral views, respectively.

##### Orthomorpha rotundicollis subrotundicollis

Taxon classificationAnimaliaPolydesmidaParadoxosomatidae

(Attems, 1937), var. nov.

82055A1B-EBCB-55EE-8924-3B7F54B83414

Figs 9 , 10 , 11

###### Material examined.

1 ♂ (ZMUMRd 4209), 1 ♀ (ZMUMRd 4201), Vietnam, Gia Lai Province, Kon Ka Kinh National Park, near the village of Krong, 14°18'03"N, 108°26'42"E, 600 m a.s.l., mixed tropical forest, on forest floor, night time, 9.V.2017; 1 ♂ (ZMUM Rd 4210), same locality, forest near the Park’s headquarters, mixed tropical forest on hill slope, 14°11'34"N, 108°19'23"E, 1,200 m a.s.l., on forest floor, daytime, 29.V.2017; 2 ♂ (ZMUM Rd 4284, Rd 4285), same locality, on log; 1 ♀ (ZMUM Rd 4283), same locality, 14°18'24"N, 108°27'13"E, 750 m a.s.l., on forest floor, daytime, 12.V.2017, all leg. I. Semenyuk.

###### Name.

To emphasize the strong similarity to the typical *O. rotundicollis* (Attems, 1937). Normally, no Latin names are to be applied to varieties as infrasubspecific categories, but because this new variety had first been qualified and described as a new species based on purely morphological grounds before the molecular evidence showed it to be the same as *O. rotundicollis*, we allot it the previously chosen name *subrotundicollis* and treat it separately in our analyses, key, and map.

###### Diagnosis.

Using the latest key (Likhitrakarn et al. 2011), this new variety keys out and seems to be especially similar to the typical *O. rotundicollis*, but it differs in the smaller size (up to 23 mm (♂) or 29 mm (♀) long and 2.9–3.2 mm (♂) or 3.3–3.9 mm (♀) wide, respectively), and the pleurosternal carinae being complete crests until segment 7 (♂) or 5 (♀), each crest supplied with an evident sharp denticle caudally, thereafter increasingly strongly reduced until segment 16 (♂, ♀), coupled with tarsal brushes being traced until ♂ legs 11.

###### Description.

Length 20.0–23.0 mm (♂) and 21.5–29.0 mm (♀), width of midbody pro- and metazonae 1.7–1.9 and 2.9–3.2 mm (♂) or 2.3–2.6 and 3.3–3.9 mm (♀), respectively.

**Figure 9.**
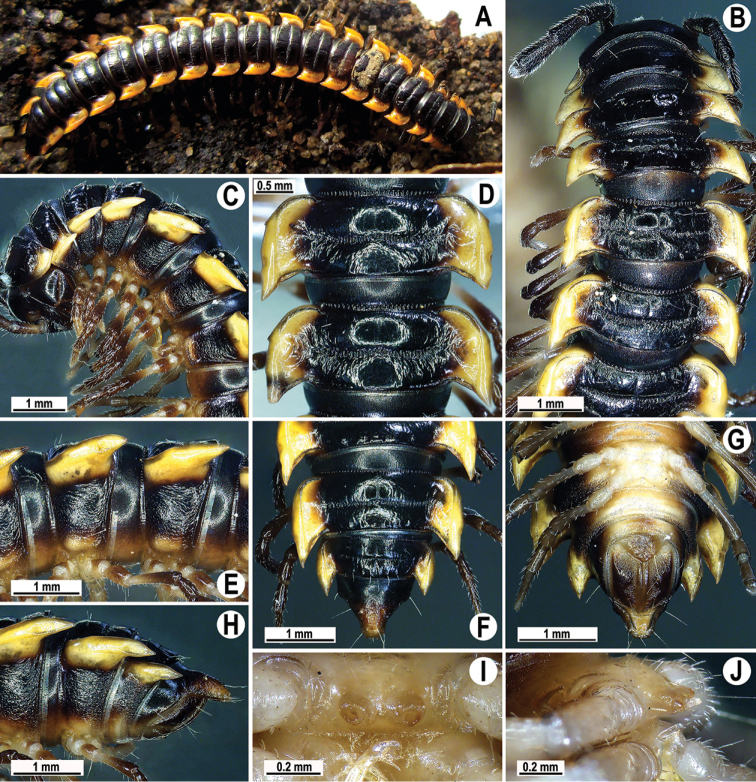
*Orthomorpha rotundicollis* (Attems, 1937), *subrotundicollis* var. nov., ♂ **A** habitus and live colouration **B, C** anterior part of body, dorsal and lateral views, respectively **D, E** segments 10 and 11, dorsal and lateral views, respectively **F–H** posterior part of body, dorsal, ventral and lateral views, respectively **I, J** sternal cones between coxae 4, subcaudal and sublateral views, respectively.

Colouration of live animals blackish (Fig. 9A), paraterga and epiproct contrasting yellow-orange, head and antennae dark brownish, legs pale brownish; colouration in alcohol, after one year of preservation, blackish or faded to black-brown (Fig. 9B–H), paraterga and epiproct pale whitish yellow to light yellow, legs and sterna light yellow to pale brown, antennae light yellow to dark brownish distally.

Clypeolabral and vertigial regions sparsely setose; epicranial suture distinct. Antennae rather short (Fig. 9A, B), reaching behind body segment 3 when stretched dorsally, with a pair of lighter, yellowish, oblong spots above antennal sockets. In width, head < collum < segment 3 < 4 < < 2 < 5–17 (♂, ♀), body gently and gradually tapering thereafter. Collum with three transverse rows of setae: 3+3 in anterior, 2+2 in intermediate, and 3+3 in posterior row; a very faint incision laterally in posterior 1/3; caudal corner of paraterga a minute, dentiform, slightly declined knob not drawn behind rear margin.

Tegument smooth and shining, prozonae finely shagreened, metaterga smooth and delicately rugulose, leathery; surface below paraterga finely microgranulate. Postcollum metaterga each with two transverse rows of setae: anterior (pre-sulcus) row with 2+2 setae traceable at least as insertion points when setae broken off; posterior (postsulcus) row with 3+3 setae borne on minute to small tubercles growing a little larger laterally, in addition to fully obliterated knobs with insertion points of abraded setae near axial line. Tergal setae long, strong, slender, ca. 1/3 of metatergal length. Axial line visible on collum and both on following pro- and metazonae.

Paraterga 2 broad, anterior edge angular, lateral edge with two small, but evident incisions in anterior 1/3; posterior edge slightly oblique (Fig. 9B, C). Following paraterga strongly developed (Fig. 9B–H), especially well so in ♂, slightly upturned caudally, all lying below dorsum, set at ca. upper 1/3 of midbody height, subhorizontal, caudal corner almost or fully pointed, increasingly spiniform and produced behind rear tergal margin; anterior edge well-developed, slightly convex to rounded, bordered and fused to callus. Lateral edge of paraterga with two small, but evident incisions, one in anterior 1/3, the other in posterior 1/3 on following poreless segments, but only one (at front 1/4) on following pore-bearing segments. Calluses on paraterga 2–4 delimited by a sulcus only dorsally, on following paraterga both dorsally and ventrally, rather narrow, modestly enlarged in pore-bearing segments, thinner in poreless ones (Fig. 9B–H). Posterior edge of paraterga clearly concave, especially well so in segments 17–19 (Fig. 9F). Ozopores evident, lateral, lying in an ovoid groove at ca. 1/3 in front of caudal corner. Transverse sulcus usually distinct (Fig. 9B, C, F), complete on metaterga 5–18, incomplete on segments 4 and 19 (♂, ♀), deep, line-shaped, nearly reaching the bases of paraterga, evidently ribbed at bottom. Stricture between pro- and metazona wide, clearly ribbed at bottom down to base of paraterga (Fig. 9B, C, F). Pleurosternal carinae complete crests with a sharp caudal tooth on segments 2–7 (♂) or 2–5 (♀), a front bulge and a caudal tooth until segment 10, each with a small sharp tooth caudally, thereafter tooth increasingly strongly reduced until segment 16 (♂, ♀) (Fig. 9C, E, H).

**Figure 10.**
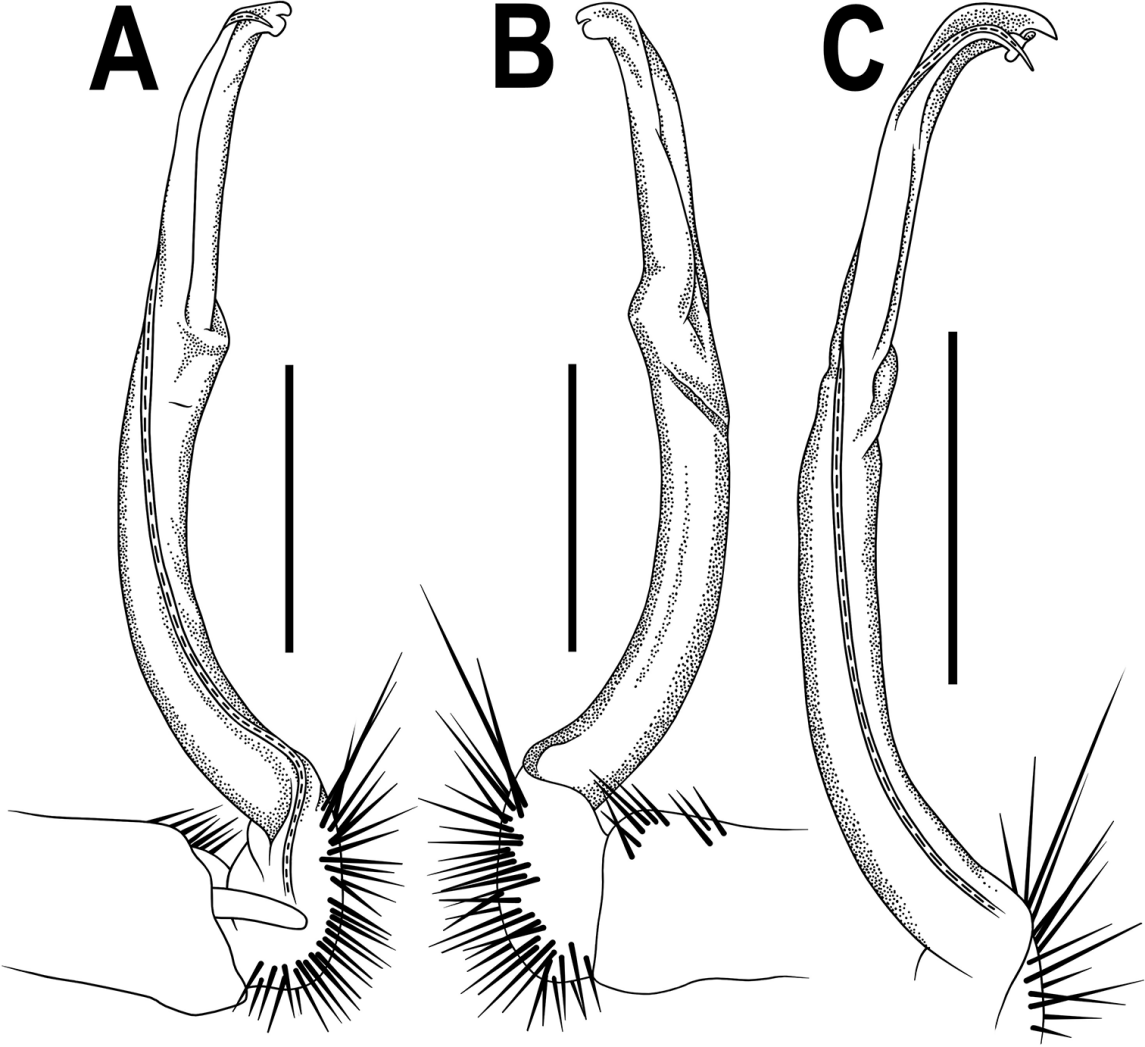
*Orthomorpha rotundicollis* (Attems, 1937), *subrotundicollis* var. nov., ♂, left gonopod **A, B** mesal and lateral views, respectively **C** distal part, submesal view. Scale bars: 0.2 mm.

Epiproct (Fig. 9F–H) conical, rounded dorsoventrally, with two evident apical papillae; tip subtruncate; pre-apical papillae evident, lying rather close to tip. Hypoproct roundly subtriangular, setiferous knobs at caudal edge evident and well-separated.

Sterna sparsely setose, without modifications except for two rather large, fully separated, setose sternal cones between ♂ coxae 4 (Fig. 9I, J). A paramedian pair of evident tubercles in front of gonopod aperture. Legs moderately long and slender; midbody ones ca. 1.1–1.2 (♂) or 0.8–0.9 (♀) times as long as body height, prefemora without modifications, ♂ tarsal brushes present until legs 11.

**Figure 11.**
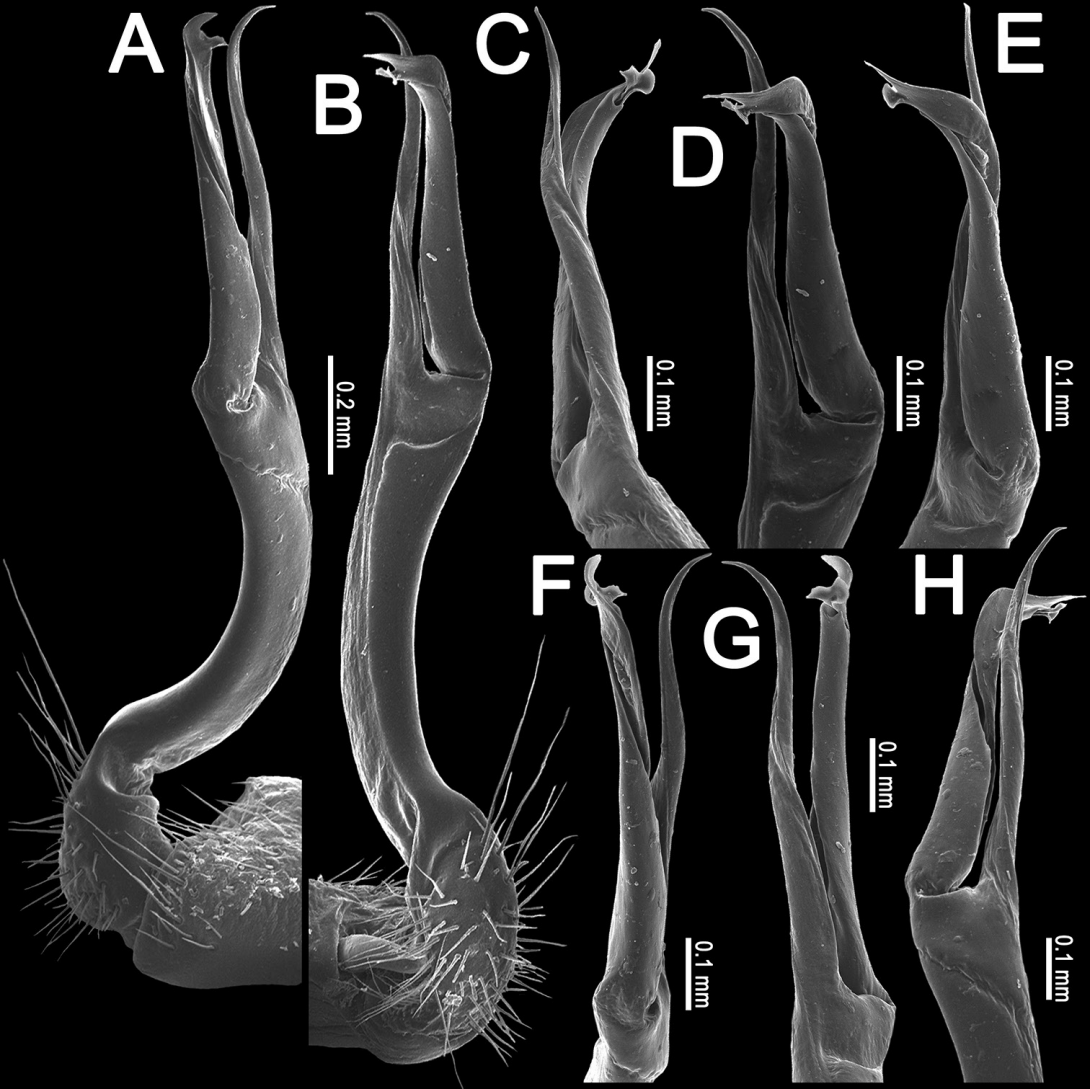
*Orthomorpha rotundicollis* (Attems, 1937), *subrotundicollis* var. nov., ♂, left gonopod **A, B** lateral and mesal views, respectively **C–H** distal part, caudal, mesal, suboral, oral, caudal and sublateral views, respectively.

Gonopods (Figs 10, 11) simple. Coxa long and slender, with several setae distoventrally. Prefemur densely setose, nearly 3 times shorter than femorite + “postfemoral” part. Femorite very slender, moderately curved, very slightly enlarged distad, “postfemoral” part demarcated by an oblique lateral sulcus; tip of solenophore small, trifid, with two subequal denticles (one lower terminal, the other middle) and a broader upper, sharp, subterminal lobule.

###### Remarks.

Based on morphological characters alone (see Diagnosis), this variety had first been qualified and distinguished as a full new species before the molecular analyses unequivocally showed it to be genetically the same as *O. rotundicollis*. Indeed, the average genetic distance (Di) of *subrotundicollis* from the typical, morphologically closest and sympatric *O. rodundicollis* is null (Table 2). In one of the phylograms (Fig. 20), *subrotundicollis* falls out between the two individuals of *rotundicollis*, and overall the genetic variation is strikingly scant. This is one of the direct consequences of the molecular analyses accepted in our study.

Adults of this variety were found only in May during a short expedition to Kon Ka Kinh National Park, as part of IS’ research on the diversity, biology, and ecology of millipedes in Vietnam. The previous trip to the same area in 2016 failed in finding this species. In 2017, millipedes were very abundant in many types of forest ranging from native to highly disturbed ones, up to even forestless hills with farmed crops. The activity was mainly noted in the night time in forest, but walking millipedes appeared even in daylight in open areas. Mating was recorded in May.

**Table 2.** Average genetic distances (Di) among the available taxa, the matrix constructed using Kimura’s two-parametric/pairwise model of nucleotide replacements that suggests a considerable prevalence of transitions over transversions in mitochondrial DNA. Old information is available in Nguyen et al. (2017, 2018) and GenBank, the new one is contained in Table 1.

		**1**	**2**	**3**	**4**	**5**	**6**	**7**	**8**	**9**	**10**	**11**	**12**	**13**	**14**	**15**	**16**	**17**	**18**	**19**	**20**	**21**	**22**
1	*Orthomorpha coarctata* Chuzhou																						
2	*O. coarctata Thac Mai*	0.004																					
3	*Antheromorpha pumatensis* Nghe An	0.153	0.146																				
4	*A. festiva* Dak Lak	0.210	0.202	0.203																			
5	*Tylopus crassipes* Lao Cai	0.261	0.270	0.192	0.265																		
6	*Oxidus gracilis* Ontario	0.227	0.235	0.242	0.253	0.209																	
7	*O. riukiarius* Okinawa	0.171	0.165	0.184	0.249	0.229	0.144																
8	*Hylomus enghoffi* Phong Nha	0.249	0.257	0.250	0.261	0.330	0.227	0.279															
9	*H. cervarius* Sa Pa	0.170	0.177	0.164	0.243	0.270	0.241	0.198	0.158														
10	*H. proximus* Sa Pa	0.205	0.212	0.235	0.261	0.206	0.284	0.291	0.200	0.174													
11	*Oxidus gigas* Duc Xuan	0.191	0.199	0.211	0.200	0.204	0.130	0.145	0.259	0.233	0.244												
12	*Piccola odontopyga* Bi Duop	0.164	0.170	0.198	0.217	0.201	0.199	0.141	0.265	0.204	0.221	0.179											
13	*Orthomorphoides setosus* Bi Duop	0.176	0.183	0.198	0.218	0.171	0.227	0.199	0.273	0.249	0.241	0.178	0.128										
14	*Orthomorpha scabra* Bi Duop	0.158	0.164	0.140	0.233	0.243	0.199	0.205	0.191	0.177	0.219	0.178	0.183	0.170									
15	*O. glandulosa* Quang Nam	0.164	0.171	0.194	0.257	0.262	0.259	0.247	0.235	0.199	0.229	0.229	0.170	0.177	0.116								
16	*O. scabra* Kon Ka Kinh	0.158	0.164	0.140	0.233	0.243	0.199	0.205	0.191	0.177	0.219	0.178	0.183	0.170	0.000	0.116							
17	O. scabra var. grandis Bidoup Nui Ba	0.158	0.164	0.140	0.233	0.243	0.199	0.205	0.191	0.177	0.219	0.178	0.183	0.170	0.000	0.116	0.000						
18	*O. vietnamica* Kon Ka Kinh	0.170	0.177	0.166	0.249	0.234	0.213	0.220	0.220	0.190	0.234	0.191	0.170	0.170	0.018	0.116	0.018	0.018					
19	*O. arboricola* Hon Ba	0.164	0.170	0.190	0.224	0.261	0.243	0.236	0.205	0.198	0.233	0.226	0.184	0.170	0.076	0.046	0.076	0.076	0.076				
20	O. rotundicollis var. subrotundicollis Kon Ka Kinh	0.152	0.158	0.159	0.204	0.173	0.204	0.198	0.219	0.172	0.250	0.221	0.121	0.158	0.115	0.148	0.115	0.115	0.104	0.141			
21	*O. rotundicollis* Thac Mai	0.152	0.158	0.159	0.204	0.173	0.204	0.198	0.219	0.172	0.250	0.221	0.121	0.158	0.115	0.148	0.115	0.115	0.104	0.141	0.000		
22	*O. rotundicollis* Cat Tien	0.152	0.158	0.159	0.204	0.173	0.204	0.198	0.219	0.172	0.250	0.221	0.121	0.158	0.115	0.148	0.115	0.115	0.104	0.141	0.000	0.000	
23	*O. caramel* Kon Ka Kinh	0.158	0.164	0.166	0.234	0.201	0.241	0.219	0.218	0.185	0.241	0.243	0.139	0.164	0.121	0.105	0.121	0.121	0.110	0.099	0.032	0.032	0.032

##### Orthomorpha caramel sp. nov.

Taxon classificationAnimaliaPolydesmidaParadoxosomatidae

77D6A3E3-5F28-5A71-9541-BBBA5DFCF21B

http://zoobank.org/0668527A-B7A6-4EE1-B5CD-7BA8B49BC540

Figs 12 , 13 , 14

###### Type material.

***Holotype*** ♂ (ZMUM Rd 4197), Vietnam, Gia Lai Province, Kon Ka Kinh National Park, forest near the village Krong, 14°18'03"N, 108°26'42"E, 600 m a.s.l., mixed tropical forest on slopes to a small stream, on tree trunk, daytime, 13.V.2017, I. Semenyuk leg.

***Paratypes*.** 1 ♂ (ZMUM Rd 4198), same locality, together with holotype; 1 ♂ (ZMUM), same locality, 14°17'46"N, 108°26'41"E, 750 m a.s.l., disturbed forest with dominating bamboo on hills, on tree trunk, daytime 18.V.2017, all leg. I. Semenyuk.

###### Name.

A noun in apposition, to emphasize the general caramel colouration of the animals.

###### Diagnosis.

Using the latest key (Likhitrakarn et al. 2011), distinguished from all known congeners by the tip of the solenophore being very faintly bifid, with a nearly smooth terminal lobe bearing a minute lobule at the base; in the gonopod structure it is similar to *O. tuberculifera* Likhitrakarn, Golovatch & Panha, 2011, but differs in the rather smooth and shining dorsal tegument devoid of tubercles, coupled with the particular colouration.

###### Description.

Length of holotype 36.5 mm (♂), width of midbody pro- and metazonae 2.8 and 4.2 mm, respectively. Paratype 31.5–34.5 mm long, 2.6–2.8 and 4.0–4.1 mm wide on midbody pro- and metazonae, respectively.

**Figure 12.**
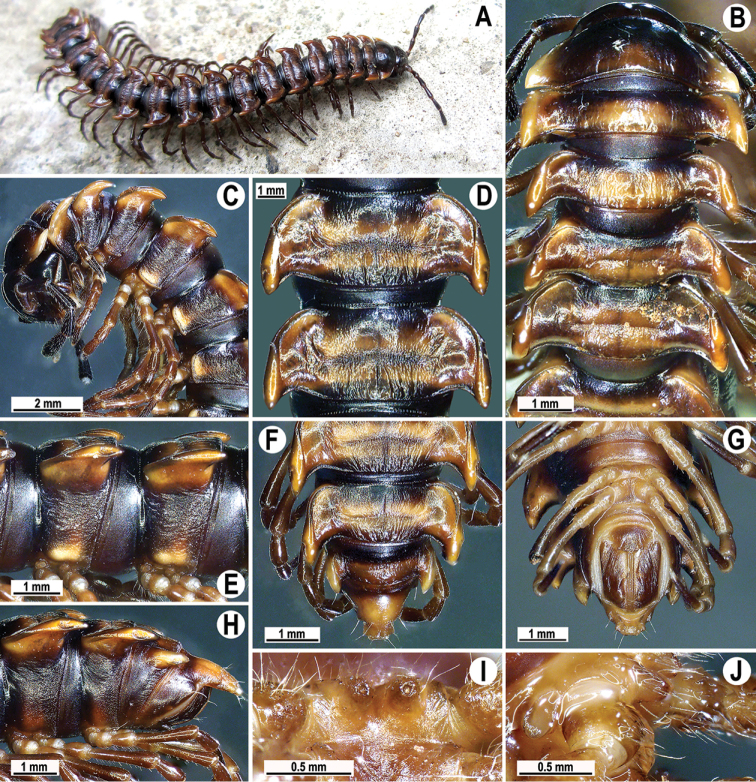
*Orthomorpha caramel* sp. nov., ♂ holotype **A** habitus, live colouration **B, C** anterior part of body, dorsal and lateral views, respectively **D, E** segments 10 and 11, dorsal and lateral views, respectively **F–H** posterior part of body, dorsal, ventral and lateral views, respectively **I, J** sternal cones between coxae 4, subcaudal and sublateral views, respectively.

Colouration of live animals dark chocolate brown (Fig. 12A); metaterga, paraterga and epiproct caramel in colour; head, antennae and legs dark brownish; colouration in alcohol after one year of preservation chocolate brown or faded to light brownish (Fig. 12B–H); metaterga, paraterga and epiproct caramel to light brownish in colour; legs and sterna light brown to pale yellow; head and antennae dark brownish to brown.

Clypeolabral region densely setose, vertigial region sparsely so; epicranial suture distinct. Antennae rather long (Fig. 12A), reaching the end of body segment 3 when stretched dorsally. In width, head < segment 3 = 4 < collum < segment 5 < 2 < 6–17, body gently and gradually tapering thereafter. Collum with three transverse rows of setae: 4+4 in anterior, 2+2 in intermediate, and 3+3 in posterior row; a slight furrow laterally in posterior 1/3; caudal corner of paraterga very narrowly rounded, slightly upturned, but not drawn behind rear margin (Fig. 12B, C).

Tegument shining, prozonae finely shagreened, metaterga rugose to rugulose, surface below paraterga finely microgranulate and rugulose. Postcollum metaterga each with two transverse rows of short, mostly abraded setae traceable only as insertion points, short wrinkles or minute tubercles: anterior (pre-sulcus) row with 2+2 mostly abraded setae traceable only as insertion points; posterior (postsulcus) row with 3+3 setae borne on minute tubercles or short wrinkles. Tergal setae long, slender, ca. 1/3 of metatergal length. Axial line rather clear, especially so on metazonae.

Paraterga very strongly developed (Fig. 12B–H), mostly upturned, all lying below dorsum, set at ca. upper 1/4 of midbody height, nearly level with dorsum on segments 15–18, caudal corner always spiniform and narrowly rounded, extending beyond rear tergal margin; in lateral view, paraterga thinner in poreless segments and modestly enlarged in pore-bearing ones.

Paraterga 2 broad, anterior edge evidently convex, lateral edge with one larger incision in anterior 1/3, one smaller, but evident incision in the middle and a faint furrow at posterior 1/3; posterior edge oblique (Fig. 12B, D, F). Anterior edges of following paraterga strongly and regularly rounded, lateral edge with only one small, but evident incision in anterior 1/3, posterior edge clearly concave, especially strongly emarginate in segments 17–19 (Fig. 12F–H). Calluses on paraterga 2–4 strongly delimited by a sulcus only dorsally, on following paraterga both dorsally and ventrally.

Ozopores evident, lateral, lying inside an ovoid groove at ca. 1/4 in front of caudal corner. Transverse sulcus usually distinct (Fig. 12C, E, H), complete on metaterga 5–18, incomplete and nearly wanting on segments 4 and 19, wave-shaped, rather shallow, nearly reaching the bases of paraterga, ribbed at bottom. Stricture between pro- and metazona rather wide and deep, beaded at bottom down to base of paraterga (Fig. 12B, D, F). Pleurosternal carinae complete crests with a sharp caudal tooth on segments 2–7(8), thereafter bulged anteriorly and with a small, sharp, caudal tooth on segments 8–10, the tooth gradually reduced into small, caudally roughly granulate crests until segment 12 (Fig. 12C, E, H). Epiproct (Fig. 12F, G) conical, flattened dorsoventrally, with two evident apical papillae; tip subtruncate; pre-apical papillae small, but evident, lying rather close to tip. Hypoproct pentagonal, setiferous knobs at caudal edge evident and well-separated.

**Figure 13.**
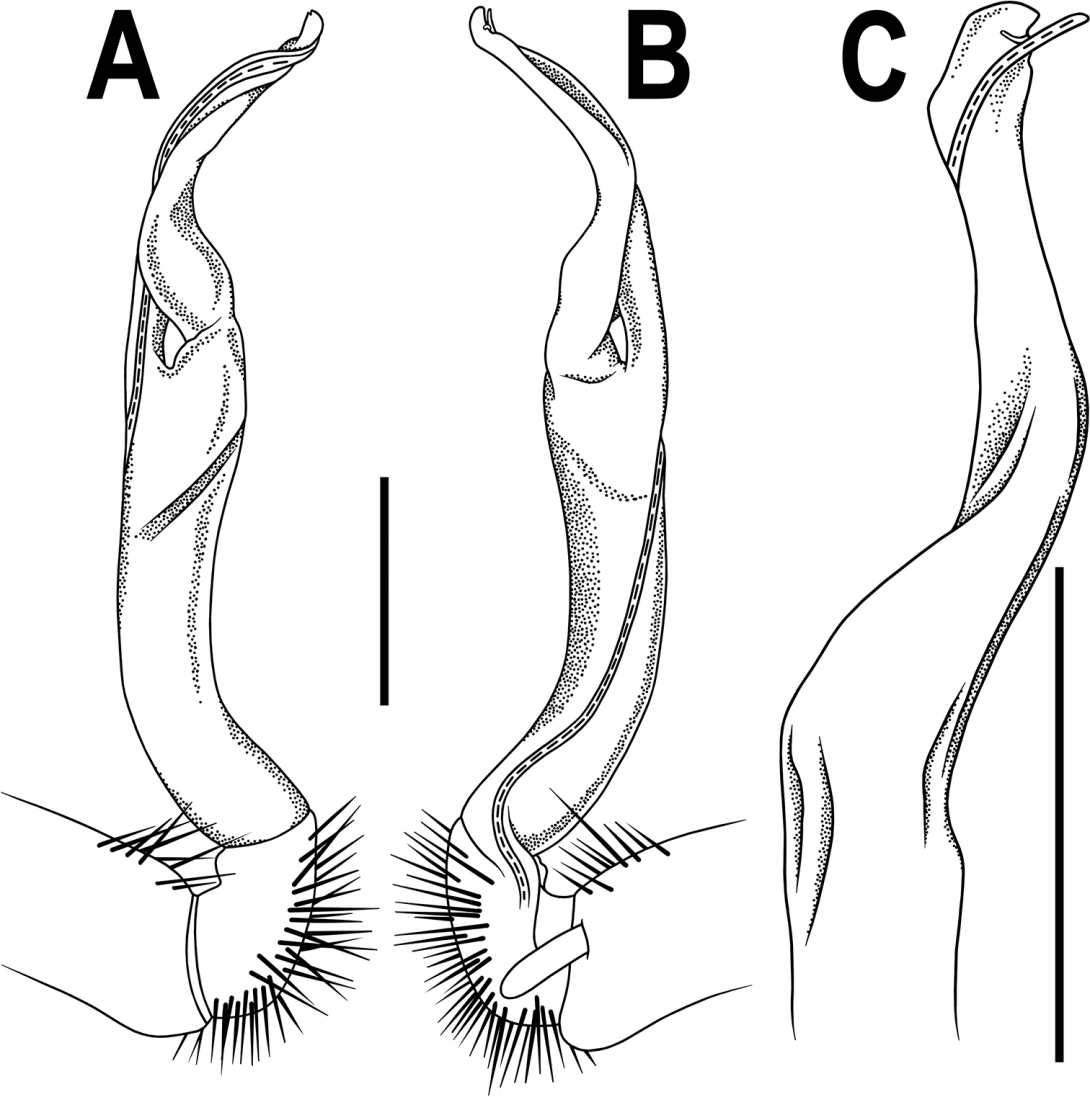
*Orthomorpha caramel* sp. nov., ♂ holotype, right gonopod **A, B** lateral and mesal views, respectively **C** distal part, suboral view. Scale bars: 0.5 mm

Sterna sparsely setose, without modifications except for two rather large and long, fully separated, sternal cones between ♂ coxae 4 (Fig. 12I, J). A paramedian pair of evident tubercles in front of gonopod aperture. Legs long and slender, midbody ones ca. 1.2–1.4 times as long as body height, prefemora without modifications, ♂ tarsal brushes present until legs 15.

**Figure 14.**
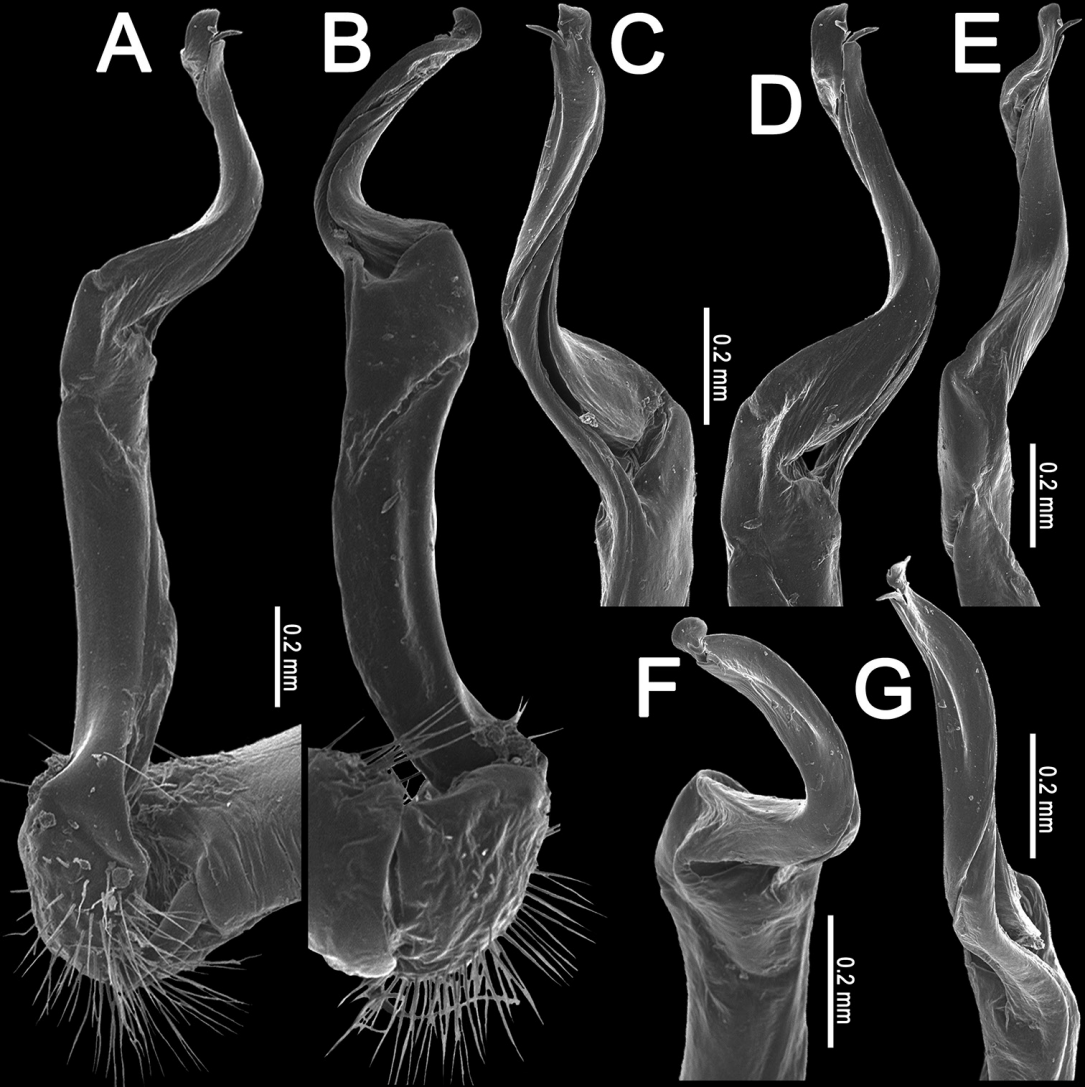
*Orthomorpha caramel* sp. nov., ♂ holotype, right gonopod **A, B** mesal and lateral views, respectively **C–G** distal part, caudal, mesal, oral, submesal and subcaudal views, respectively.

Gonopods (Figs 13, 14) simple. Coxa slender and long, with several setae distoventrally. Femorite ca. 2 times as long as prefemoral (= strongly setose) part. Femorite slender, suberect to slightly curved, “postfemoral” portion demarcated by an oblique lateral sulcus; solenophore moderately twisted and curved, tip of solenophore very faintly bifid, with a nearly smooth terminal lobe bearing a minute lobule at base; solenomere long and flagelliform, as usual.

###### Remarks.

The biology and behaviour of this species are very similar to those of *O. vietnamica* sp. nov. During field observations in May 2017, millipedes occurred mainly on tree trunks. Mating was also recorded, but no females were collected.

##### Orthomorpha vietnamica sp. nov.

Taxon classificationAnimaliaPolydesmidaParadoxosomatidae

F46385BD-1947-58D1-AC4F-1147AB6154DD

http://zoobank.org/D9F27912-213D-4AEB-AE74-38FFFFA0F66B

Figs 15 , 16

###### Type material.

***Holotype***: ♂ (ZMUM Rd 4199), Vietnam, Gia Lai Province, Kon Ka Kinh National Park, near the village of Krong, 14°18'03"N, 108°26'42"E, 600 m a.s.l., mixed tropical forest, on tree trunk, night time, 9.V.2017, I. Semenyuk leg.

***Paratype***: 1 ♀ (ZMUM Rd 4200), same locality, together with holotype.

###### Name.

Adjective to refer to the country of origin.

###### Diagnosis.

This species seems to be especially similar to *O. caramel* sp. nov., but differs from all congeners (Likhitrakarn et al. 2011) in the presence of a conspicuous, densely setose, rounded tubercle (Fig. 16, cxp) on the gonocoxa and the evident trifid tip of the solenophore, as well as the ♂ tarsal brushes showing until segment 16, coupled with the pleurosternal carinae present as complete crests with a sharp caudal tooth on segments 2–4.

###### Description.

Length 27.5 mm (♂) or 29.5 mm (♀), width of midbody pro- and metazonae 2.0 and 3.0 mm (♂) or 2.7 and 3.6 mm (♀), respectively.

**Figure 15.**
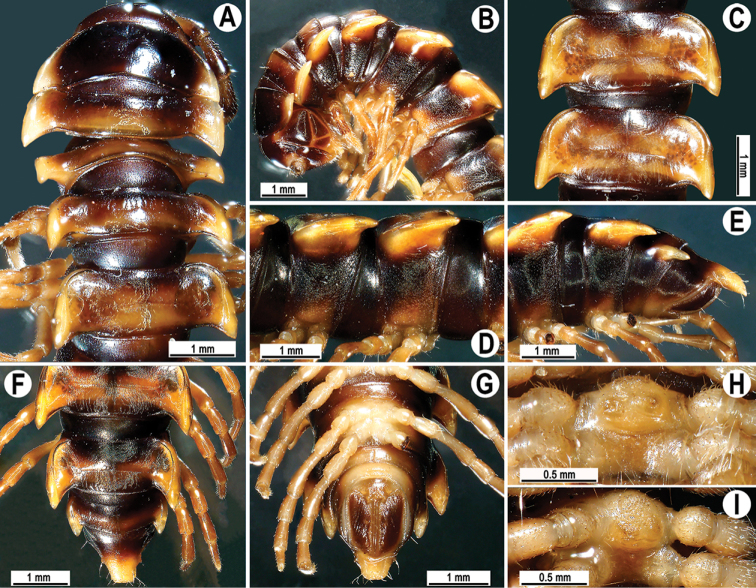
*Orthomorpha vietnamica* sp. nov., ♂ holotype **A, B** anterior part of body, dorsal and lateral views, respectively **C, D** segments 10 and 11, dorsal and lateral views, respectively **E–G** posterior part of body, lateral, dorsal and ventral views, respectively **H, I** sternal cones between coxae 4, subcaudal and sublateral views, respectively.

Colouration of alcohol material after one year of preservation dark brown (Fig. 10B–H); metaterga brown to light brownish; paraterga and epiproct light yellow to yellowish; legs and sterna light brown to pale yellow; head, collum, and antennae dark brownish to brown. Bases of paraterga marbled (Fig. 15C).

All characters as in *O. caramel* sp. nov., except as follows: clypeolabral region sparsely setose, vertigial region bare. Antennae rather short, reaching behind body segment 3 (♂, ♀) when stretched dorsally. In width, head < collum < segment 3 < 4 < 5 < 2 < 6–17 (♂) or head < collum < segment 3 < 4 < 2 < 5–17 (♀), body gently and gradually tapering thereafter.

Tegument rather dull, prozonae finely shagreened, metaterga rugose to rugulose, surface below paraterga finely microgranulate and faintly rugulose (Fig. 15A–F).

Axial line visible on collum and both on following pro- and metazonae.

Transverse sulcus usually distinct (Fig. 15C, E, H), complete on metaterga 5–18, incomplete and nearly wanting on segments 4 and 19, nearly line-shape, rather shallow, almost reaching the bases of paraterga, at most very faintly beaded at bottom.

Pleurosternal carinae complete crests with a sharp caudal tooth on segments 2–4 (♂) (Fig. 15B) or nearly complete crests with a sharp caudal tooth on segment 4 (♀), thereafter bulged anteriorly and with a small sharp caudal tooth until segment 8, the tooth gradually reduced into small, caudally roughly granulate crests until segment 12 (♂, ♀). Epiproct (Fig. 15F, G) conical, flattened dorsoventrally, tip subtruncate; with two evident, sharp, apical teeth directed ventrocaudally (♂, ♀); pre-apical papillae very small, located close to tip. Hypoproct roundly subtriangular, setiferous knobs at caudal edge evident and well-separated (Fig. 15G).

Sterna sparsely setose, without modifications except for an evident, rounded, sparsely setose bulge directed anteroventrally between ♂ coxae 4 (Fig. 15I, J). A paramedian pair of evident, high tubercles in front of gonopod aperture. Legs rather long and slender, midbody ones ca. 1.2–1.3 (♂) or 0.8–0.9 (♀) times as long as body height, prefemora without modifications, ♂ tarsal brushes present until legs of segment 16.

**Figure 16.**
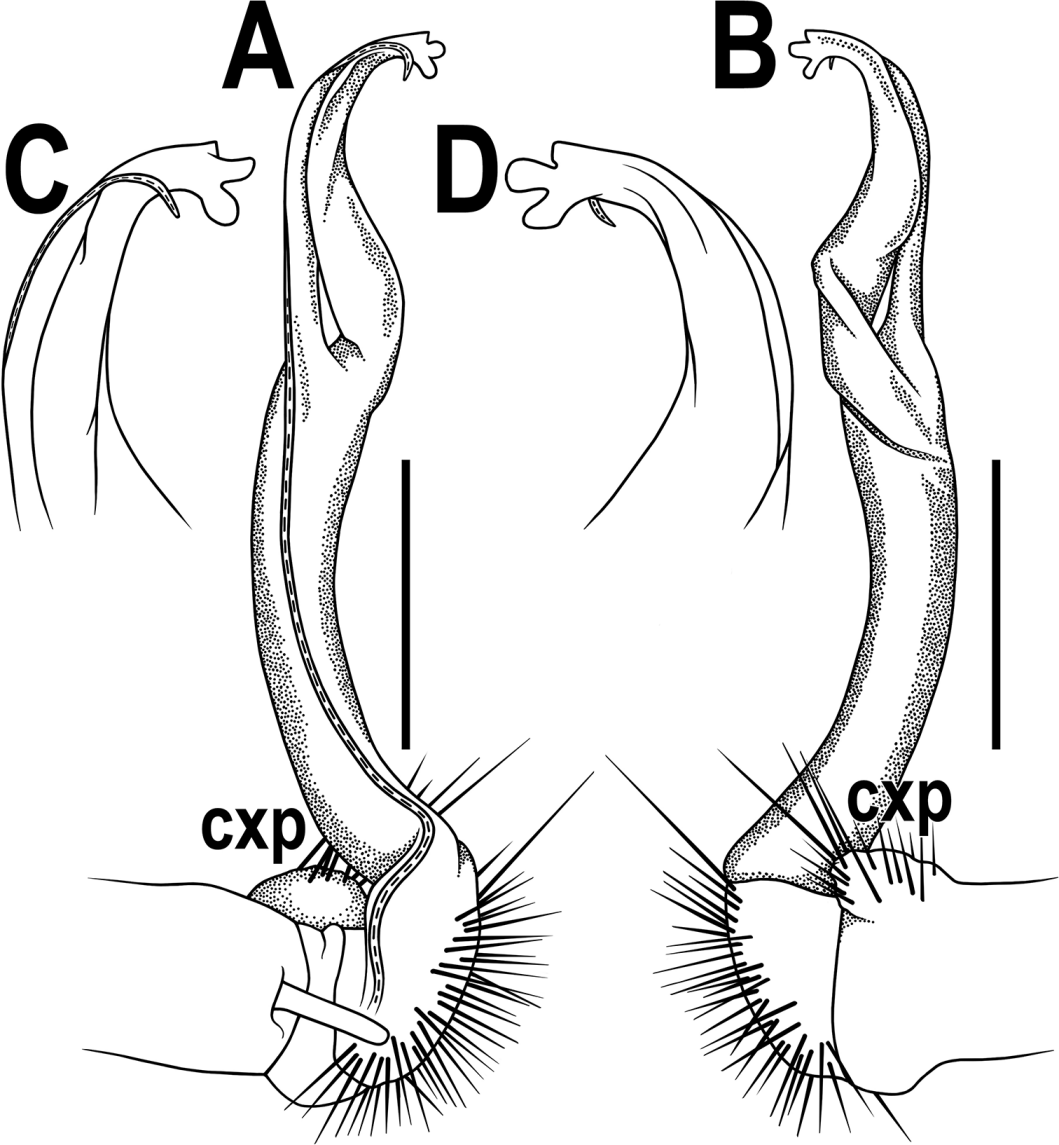
*Orthomorpha vietnamica* sp. nov., ♂ holotype, left gonopod **A, B** mesal and lateral views, respectively **C, D** tip of gonopod, submesal and sublateral views, respectively. Abbreviation: **cxp**, coxal tubercle. Scale bars: 0.5 mm.

Gonopods (Fig. 16) somewhat more complex than usual. Coxa long and slender, with a conspicuous, high, laterally densely setose, rounded tubercle (Fig. 16A, B, cxp). Femorite ca. 2 times as long as prefemoral (= strongly setose) part. Femorite slender, slightly curved and enlarged distad, “postfemoral” portion demarcated by an oblique lateral sulcus; solenophore with a tridentate tip, two subequal, rounded lobules (middle and ventral) and a shorter apicodorsal lobule; solenomere long and flagelliform, as usual.

###### Remarks.

We assume the apicodorsal lobule of the solenophore to be broken off, considering the structure of the solenophore tip in the other *Orthomorpha* spp.

The ecology of this species is very similar to that of *O. caramel* sp. nov. These two species are syntopic and share the same microhabitats.

##### Orthomorpha scabra grandis

Taxon classificationAnimaliaPolydesmidaParadoxosomatidae

Jeekel, 1964, var. nov.

90ECC702-8584-5DDF-A2A8-F6E0BF0BE283

Figs 17 , 18

###### Material examined.

4 ♂ (ZMUM Rd 4268, Rd 4266, Rd 4267, Rd 4269), Vietnam, Lam Dong Province, Bidoup Nui Ba National Park, Mount Bidoup, 12°05'58"N, 108°39'30"E, 2,000 m a.s.l., mixed cloudy mossy forest on mountain top, on forest floor, night time, 16.VI.2018, I. Semenyuk leg.

###### Name.

Adjective to emphasize the unusually large size of the animals. Normally, no Latin names are to be applied to varieties as infrasubspecific categories, but because this new variety had first been qualified and described as a new species based on purely morphological grounds before the molecular evidence showed it to be the same as *O. scabra*, we allot it the previously chosen name *grandis* and treat it separately in our analyses, key, and map. This is also one of the direct consequences of the molecular analyses accepted in our study.

###### Diagnosis.

Distinguished from all known congeners or varieties by the particularly large size, coupled with the clearly tuberculate metaterga, caudolaterally rounded paraterga 1–14, and the relatively short gonopodal femorite.

###### Description.

Length 44–50 mm, width of midbody pro- and metazonae 3.5–3.8 and 5.2–5.6 mm wide on midbody pro- and metazonae, respectively (♂).

**Figure 17.**
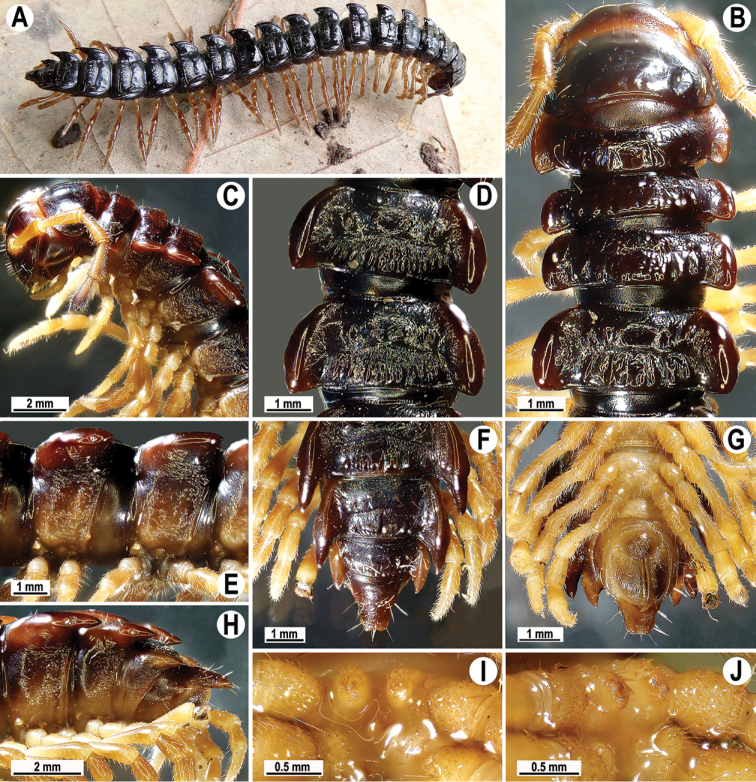
*Orthomorpha scabra* Jeekel, 1964, *grandis* var. nov., ♂ **A** habitus and live colouration **B, C** anterior part of body, dorsal and lateral views, respectively **D, E** segments 10 and 11, dorsal and lateral views, respectively **F–H** posterior part of body, dorsal, ventral and lateral views, respectively **I, J** sternal cones between coxae 4, subcaudal and sublateral views, respectively.

Colouration in alcohol after ten months of preservation dark brown (Fig. 17A–G); metaterga, paraterga and epiproct red-brown to dark brown; legs, antennae and sterna light brown to light yellow; head brownish to brown.

Clypeolabral region densely setose, vertigial region sparsely so; epicranial suture distinct. Antennae rather long (Fig. 17B), reaching the end of body segment 3 when stretched dorsally. In width, head < segment 3 < 4 < collum < segment 5 < 2 < 6–14, body gently and gradually tapering thereafter. Collum with three transverse rows of setae: 3+3 in anterior, 2+2 in intermediate, and 4+4 in posterior row; a slight furrow laterally in posterior 1/3; caudal corner of paraterga very broadly rounded, slightly upturned, but not drawn behind rear margin (Fig. 17A, B).

Tegument shining, prozonae finely shagreened, postcollum metaterga rugose to rugulose and clearly tuberculate, surface below paraterga finely microgranulate and rugulose. Postcollum metaterga each with two transverse rows of abraded setae: anterior (pre-sulcus) row with 2(3)+2(3) mostly minute tubercles, short wrinkles or traceable only as insertion points, posterior (postsulcus) row with 3–5+3–5 setae borne on low, oblong, rounded tubercles or minute knobs. Tergal setae long, slender, ca. 1/3 of metatergal length. Axial line rather clear, especially so on metazonae.

Paraterga 2 broad, anterior edge evidently convex, lateral edge with three minute incisions in anterior 1/4 to half; posterior edge oblique (Fig. 17A). Anterior edges of following paraterga regularly rounded, lateral edge without incisions, posterior edge oblique to regularly concave, especially strongly concave in segments 14–19 (Fig. 17E–G). Calluses on paraterga 2–4 strongly delimited by a sulcus only dorsally, on following paraterga both dorsally and ventrally.

Following paraterga very strongly developed (Fig. 17A–F), upturned, lying below dorsum until segment 4, following ones above dorsum, caudal corner ranging from obtuse-angular to subrectangular, always and increasingly extending behind rear tergal margin (Fig. 17A–C), from segment 15 on spiniform, long and pointed; in lateral view, paraterga thinner in poreless segments and modestly enlarged in pore-bearing ones.

Ozopores evident, lateral, lying inside an ovoid groove at ca. 1/4 in front of caudal corner. Transverse sulcus usually distinct (Fig. 17A, C, F), complete on metaterga 5–18, incomplete on segment 4, wave-shaped, rather shallow, nearly reaching the bases of paraterga, very faintly ribbed at bottom. Stricture between pro- and metazona rather wide and deep, very faintly ribbed at bottom down to base of paraterga (Fig. 17A, C, F). Pleurosternal carinae complete crests with a sharp caudal tooth on segments 2–4(8), thereafter bulged anteriorly and with a small, sharp, caudal tooth on segments 8–13, the tooth gradually reduced into small, caudally roughly granulate crests until segment 16 (Fig. 17B, D, E). Epiproct (Fig. 17F, G) conical, flattened dorsoventrally, with two evident apical papillae; tip subtruncate; pre-apical papillae very small, but traceable, lying rather close to tip. Hypoproct subtriangular, setiferous knobs at caudal edge evident and well-separated.

Sterna sparsely setose, without modifications except for two rather large and long, fully separated, sternal cones between ♂ coxae 4 (Fig. 17H, I). A paramedian pair of small tubercles in front of gonopod aperture. Legs long and slender, midbody ones ca. 1.3–1.4 times as long as body height, prefemora without modifications, ♂ tarsal brushes present until legs 7.

**Figure 18.**
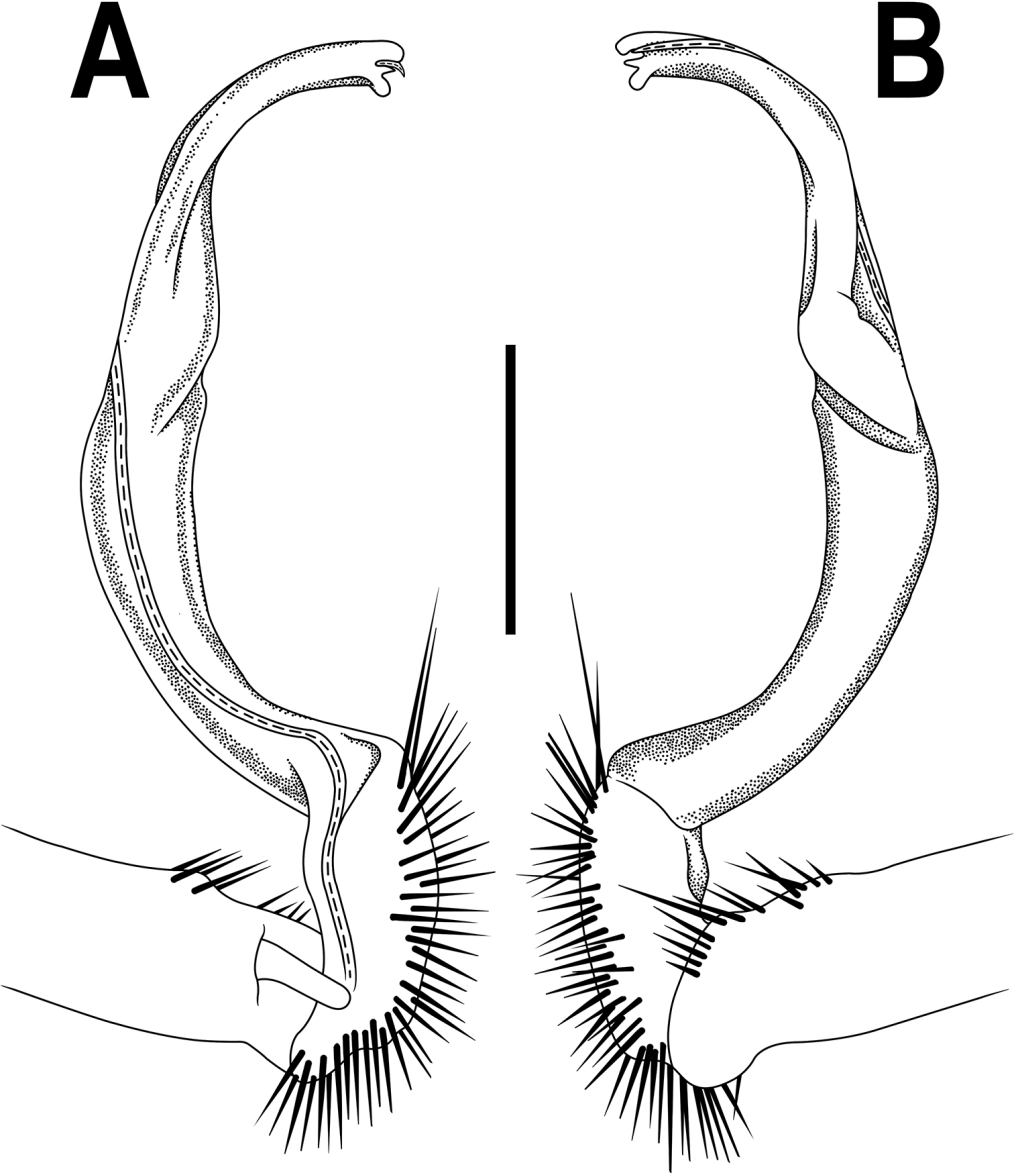
*Orthomorpha scabra* Jeekel, 1964, *grandis* var. nov., ♂, left gonopod **A, B** mesal and lateral views, respectively. Scale bar: 0.5 mm.

Gonopods (Fig. 18) simple. Coxa slender and long, with several setae distoventrally. Femorite relatively short, ca. 2 times as long as prefemoral (= strongly setose) part. Femorite slender, moderately curved, “postfemoral” portion demarcated by an oblique lateral sulcus; solenophore with a tridentate tip, middle denticle shorter than terminal tooth, but longer than a small subterminal lobule; solenomere long, flagelliform, tip barely exposed.

###### Remark.

This species was found only on Mount Bidoup in the summit area, while none of the inspected adjacent forests, even those located at the same altitudes, including a very similar forest on Mount Hon Giao, failed to reveal these millipedes. On Mount Bidoup, this species was quite abundant, occurring mainly on the forest floor, on logs and at bases of tree trunks in the night time, as well as under logs in the daytime.

#### Key to the species and varieties of *Orthomorpha* currently known to occur in Vietnam, based on ♂ characters (constructed on the key by Likhitrakarn et al. 2011).

**Table:** 

1	Colouration not strikingly contrasting, calluses being only inconspicuously paler than a dark remaining background (Figs 1A–F, 6A–H, 12A–H, 15A–G, 17A–H)	**2**
–	Colouration of metaterga more or less strongly contrasting, with very pale calluses against a very dark remaining background (Figs 3A–G, 9A–H)	**6**
2	Each metatergum at least with one, rather evident, transverse row of tubercles near caudal margin (Figs 1A–C, F, 6A–D, F, 17A–C, F)	**3**
–	Metaterga smooth and shining, at most faintly rugulose (Figs 12A–D, F, 15A–C, F)	**5**
3	Paraterga 1–14 clearly rounded caudolaterally (Fig. 17A–D)	***O. scabra grandis* var. nov.**
–	All paraterga clearly sharpened and pointed caudolaterally (Figs 1A–D, 6A–E)	**4**
4	A single sternal cone between ♂ coxae 4 (Fig. 1H, I). Distal part of gonopod evidently curved and tip bifid (Fig. 2)	***O. arboricola***
–	Two separated sternal cones between ♂ coxae 4 (Fig. 6I, J). Distal part of gonopod slightly curved and tip trifid (Figs 7, 8)	***O. scabra***
5	Pleurosternal carinae complete and high crests, each with a sharp caudal tooth on segments 2–7 (♂) (Fig. 12C, E, H). Tarsal brushes present until segment 10 (♂). Tip of gonopod very faintly bifid, with a minute lobule at base (Figs 13, 14)	***O. caramel* sp. nov.**
–	Pleurosternal carinae complete high crests, each with a sharp caudal tooth on segments 2–4 (♂, ♀) (Fig. 15B, D, E). Tarsal brushes present until segment 16 (♂). Tip of gonopod evidently trifid and coxa with a conspicuous, densely setose, rounded, distoventral tubercle (cxp) (Fig. 16A, B)	***O. vietnamica* sp. nov.**
6	Gonopod tip a single, very small, rounded lobule. Virtually no modifications between ♂ coxae 4. Pantropical	***O. coarctata***
–	Gonopod tip trifid (Figs 4, 5, 10, 11). Modifications between ♂ coxae 4 mostly present (Figs 3H, I, 9I, J)	**7**
7	Paraterga largely level with or even elevated above dorsum. Sternal cone between ♂ coxae 4 single, large. Distal part of gonopod evidently curved	***O. glandulosa***
–	All paraterga set below dorsum. Two small, separated, sternal cones between ♂ coxae 4 (Figs 3I, J, 9I, J). Distal part of gonopod slightly curved	**8**
8	Tarsal brushes present on ♂ legs 1–7. Transverse sulcus complete and distinct until metatergum 17. Sternal cone between ♂ coxae 4 very low. Gonopod telopodite very slender	***O. hydrobiologica***
–	Tarsal brushes present also past ♂ leg 10. Transverse sulcus complete and distinct until metatergum 18. Sternal cone between ♂ coxae 4 high (Figs 3I, J, 9I, J). Gonopod rather stout (Figs 4, 5, 10, 11)	**9**
9	Larger (29–38.5 (♂) or 31–40.5 mm (♀) long and 3.5–4.3 (♂) or 3.6–4.9 mm (♀) wide, respectively). Pleurosternal carinae complete crests on segments 2–4 (♂, ♀), each with an evident sharp denticle caudally, thereafter increasingly strongly reduced until segment 10 (♂). Tarsal brushes present until legs of ♂ segment 10	***O. rotundicollis***
–	Smaller (up to 23 (♂) or 29 mm (♀) long and 2.9–3.2 (♂) or 3.3–3.9 mm (♀) wide, respectively). Pleurosternal carinae complete crests until segment 7 (♂) or 5 (♀), each with an evident sharp denticle caudally, thereafter increasingly strongly reduced until segment 16 (♂, ♀). Tarsal brushes present until ♂ legs 11	***O. rotundicollis subrotundicollis* var. nov.**

## Conclusions

The phylogenetic analyses performed here are considered as supporting to the traditional morphological taxonomy since it is a single-gene topology. While it should provide some resolution to disentangling *Orthomorpha* evolutionary history, the limitations of using just one gene are evident. The more so as, due to fixation problems with the DNA, we have only been able to get approximately one-third of the COI gene. In addition, not only sympatric and closely related species, but even certain genera of Paradoxosomatidae sometimes seem to show hybridization (Decker 2018).

**Figure 19.**
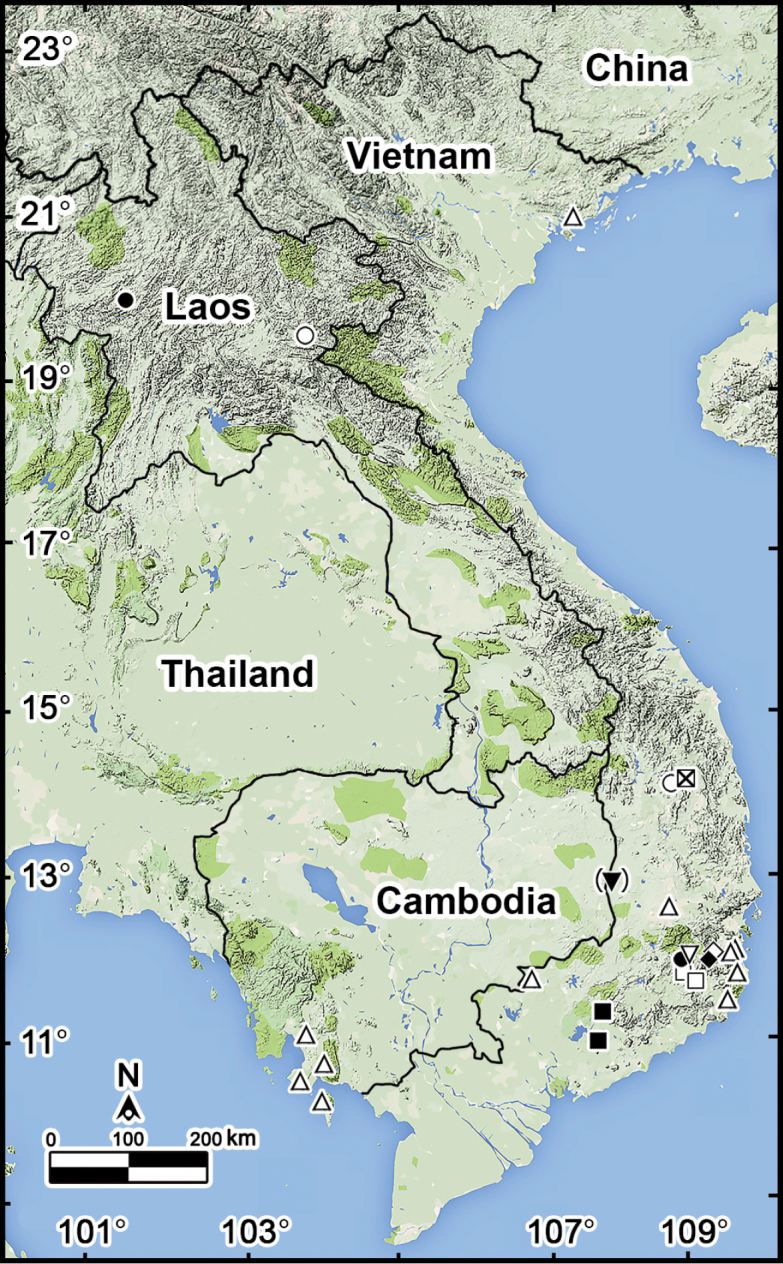
Distributions of *Orthomorpha* species (nine species or varieties, excluding the pantropical anthropochore *O. coarctata* (de Saussure, 1860)) recorded in Vietnam. **Open triangle***O. hydrobiologica* Attems, 1930 **Filled circle***O. scabra* Jeekel, 1964 **Open circle***O. scabra*, *grandis* var. nov., *O. scabra* Jeekel, 1964 and *O. rotundicollis* (Attems, 1937) **Crossed square***O. rotundicollis*, *subrotundicollis* var. nov., *O. caramel* sp. nov. and *O. vietnamica* sp. nov. **Filled Inverted triangle***O. glandulosa* (Attems, 1937) **Opened Inverted triangle***O. scabra* Jeekel, 1964 and *O. scabra, grandis* var. nov. **Open diamond***O. glandulosa* (Attems, 1937) and *O. hydrobiologica* Attems, 1930 **Filled diamond***O. glandulosa* (Attems, 1937) **Open square***O. arboricola* (Attems, 1937) and *O. rotundicollis* (Attems, 1937) **Filled square***O. rotundicollis* (Attems, 1937) and *O. coarctata* (de Saussure, 1860).

**Figure 20.**
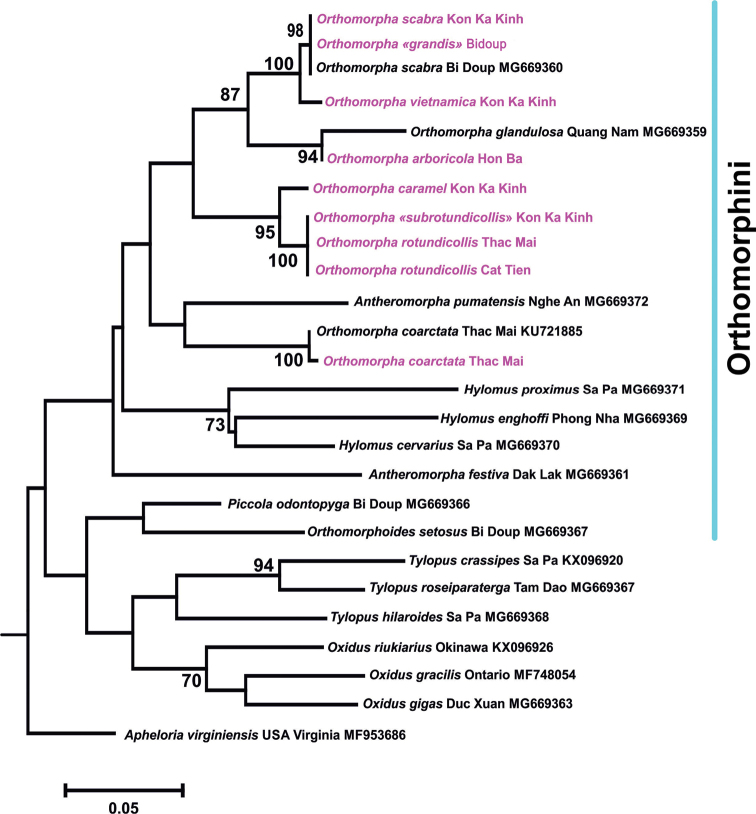
A rooted phylogram based on COI sequences obtained from the relevant literature on Paradoxosomatidae (Nguyen et al. 2017, 2018) and available in GenBank (all given in black, yet with several name updates according to modern taxonomy), combined with new haplotypes (six species and two varieties, these latter put in quotation marks and all highlighted in red). Tree topology was reconstructed using MrBayes software. The values of statistical support are given above the branches only when these exceed 65%.

As our study shows (Tables 1, 2, Fig. 20), within the tribe Orthomorphini, almost all particularly closely related species belong to the genus *Orthomorpha*. Only *O. coarctata* is set apart and appears to be closer to other genera (Table 2). This is clearly seen also in Figure 20. Again only *O. coarctata* shows a special position clustering closer to *Antheromorpha* Jeekel, 1968 and *Hylomus* Cook & Loomis, 1924 than to other formal congeners. This is hardly surprising though, because that species is often treated as the sole constituent member of a separate genus, *Asiomorpha* Verhoeff, 1939 (cf. Nguyen and Sierwald 2013). However, we prefer to stick here to the traditional, purely morphologically based concept of the Orthomorphini and *Orthomorpha*, taking most of our and all previous molecular evidence as only subordinate, provisional, and auxiliary.

Based on a different genetic marker (16S ribosomal gene), Nguyen et al. (2018) showed that *Antheromorpha* appears as sister group to *Orthomorpha* + *Nesorthomorpha*. The results of our analysis (Fig. 20) suggest, however, that *Orthomorpha* (minus *coarctata*) is sister to *Antheromorpha* + *coarctata* + *Hylomus* (NB: Nguyen et al. (2018) treated *Hylomus* as *Desmoxytes* Chamberlin, 1923).

We present here only a phylogram rooted in *Apheloria virginiensis* (Drury, 1770) (Xystodesmidae) as perhaps the closest outgroup currently available (Fig. 20). Even though the presence or absence of a root can drastically change tree topology, because our unrooted trees completely failed to alter the picture, they have been omitted. The same applies to a number of other rooted phylograms.

However, as noted above, two important taxonomic changes have been accepted following solely our molecular analyses and trees. We simply follow a cautious approach to avoid descriptions of suspicious new taxa/species. These changes concern the admittance of two morphologically distinct (especially so in body size), but genetically unsupported varieties, *O. rotundicollis subrotundicollis* var. nov. and *O. scabra grandis* var. nov. Because neither is distinguished from their typical forms by average genetic distances (Table 2), they have been allotted no taxonomic status. The other results, however, clearly suggest that more molecular and morphology-based taxonomic work should be paid to *Orthomorpha* in the future. The animals are large-sized, often vividly coloured and readily exposed, thus being easy to spot and collect. In addition, *Orthomorpha* enjoys a recent, colourful, open-access, monographic treatment with an identification key to species (Likhitrakarn et al. 2011).

In any event, although the trees both in Nguyen et al. (2017, 2018) and our present research must only be taken as provisional, they provide a basis for future phylogenetic analyses implementing additional gene regions. Further efforts, both morphology- and molecular-based, are required to reveal and refine the relationships between Orthomorphini (and not only) genera and species. At least for the time being, the Orthomorphini seems to be a monophyletic tribe of Paradoxosomatidae, whereas *Orthomorpha* remains polyphyletic and *Antheromorpha* is paraphyletic (Fig. 20).

A total of eight species and two morphologically distinct varieties of *Orthomorpha* is currently recognized in the fauna of Vietnam, including two new congeners: *O. caramel* sp. nov. and *O. vietnamica* sp. nov. Both new species come from national parks and surrounding areas, thus priority zones for the protection of biodiversity in Vietnam. Several, often two or three species co-exist in one place and are strictly syntopic, this having been observed, e.g., in the pristine tropical forest of the Kon Ka Kinh National Park (Fig. 19).

Most of the Vietnamese species tend to be restricted to the southern parts of Vietnam, except for the likely anthropochore *O. hydrobiologica* which ranges from Hong Gai, Quảng Ninh Province, northern Vietnam to southern Cambodia. The range of *O. coarctata* in Vietnam must be even greater, covering most of the country in man-made habitats. No native species seem to exist in northern Vietnam. A similar distribution pattern concerns Laos where no native *Orthomorpha* seem to populate the northern parts of the country (Likhitrakarn et al. 2014).

There can be little doubt that further new *Orthomorpha* species and/or records await discovery in Indochina, including Vietnam. This seems particularly topical in the still persisting forested areas of that huge region.

## Supplementary Material

XML Treatment for Orthomorpha arboricola

XML Treatment for Orthomorpha coarctata

XML Treatment for Orthomorpha glandulosa

XML Treatment for Orthomorpha hydrobiologica

XML Treatment for Orthomorpha rotundicollis

XML Treatment for Orthomorpha scabra

XML Treatment for Orthomorpha rotundicollis subrotundicollis

XML Treatment for Orthomorpha caramel

XML Treatment for Orthomorpha vietnamica

XML Treatment for Orthomorpha scabra grandis
